# Stability and dynamics of membrane-spanning DNA nanopores

**DOI:** 10.1038/ncomms14784

**Published:** 2017-03-20

**Authors:** Vishal Maingi, Jonathan R. Burns, Jaakko J. Uusitalo, Stefan Howorka, Siewert J. Marrink, Mark S. P. Sansom

**Affiliations:** 1 Department of Biochemistry, University of Oxford, South Parks Road, Oxford OX1 3QU, UK; 2 Department of Chemistry, Institute of Structural Molecular Biology, University College London, London WC1H 0AJ, UK; 3 Groningen Biomolecular Sciences and Biotechnology Institute and Zernike Institute for Advanced Materials, University of Groningen, Nijenborgh 7, 9747 AG Groningen, The Netherlands

## Abstract

Recently developed DNA-based analogues of membrane proteins have advanced synthetic biology. A fundamental question is how hydrophilic nanostructures reside in the hydrophobic environment of the membrane. Here, we use multiscale molecular dynamics (MD) simulations to explore the structure, stability and dynamics of an archetypical DNA nanotube inserted via a ring of membrane anchors into a phospholipid bilayer. Coarse-grained MD reveals that the lipids reorganize locally to interact closely with the membrane-spanning section of the DNA tube. Steered simulations along the bilayer normal establish the metastable nature of the inserted pore, yielding a force profile with barriers for membrane exit due to the membrane anchors. Atomistic, equilibrium simulations at two salt concentrations confirm the close packing of lipid around of the stably inserted DNA pore and its cation selectivity, while revealing localized structural fluctuations. The wide-ranging and detailed insight informs the design of next-generation DNA pores for synthetic biology or biomedicine.

## Abstract (web summary)

Although DNA nanopores are widely explored as synthetic membrane proteins, it is still unclear how the anionic DNA assemblies stably reside within the hydrophobic core of a lipid bilayer. Here, the authors use molecular dynamics simulations to reveal the key dynamic interactions and energetics stabilizing the nanopore-membrane interaction.

Cell membranes are formed by lipid bilayers and membrane proteins. The proteins carry out key biological functions, including transport of ions and other hydrophilic solutes across membranes. Tuning the function of proteins is of considerable interest in synthetic biology but challenging due to the difficulty of designing predictable protein structures. Recent studies of synthetic DNA nanomaterials have suggested an alternative route to mimic the functions of membrane proteins^1^. Unlike polypeptides, synthetic DNA structures are obtained by simple programmable self-assembly and thus have the potential to expand the toolkit of synthetic biology. However, creating such biomimetic assemblies that mimic membrane-inserted proteins requires that hydrophilic negatively charged DNA molecules are inserted into a lipid bilayer and span its hydrophobic core. An example of how a DNA nano-assembly may be inserted are DNA nanotubes (DNTs) that carry covalently attached hydrophobic anchors to form stable membrane-spanning nanopores^2^.

In general, nanopores help us to explore fundamental aspects of solute transport at the nanoscale^3^. They are also used in biotechnology^4^ and biomedicine^5^, such as in stochastic sensors^6^,^7^, and for the controlled translocation of proteins^8^ or nucleic acids^9^,^10^ across membranes. Of especial importance is their use in next-generation sequencing of DNA^11^. Reflecting this scientific and technological interest, nanopores have been made by a range of materials^12^ including natural biopolymers, such as proteins^13^ and peptides^14^, and also synthetic organic molecules^15^ and carbon nanotubes^16^,^17^.

DNTs are the most recent class of artificial membrane pores^18^,^19^,^20^. They are formed via self-assembly^18^ using the principles of DNA origami^21^. DNTs can be designed *de novo* with a wider range of architectures and functionalities than offered by protein nanopores. A number of DNT designs have been explored^18^,^19^,^22^,^23^,^24^ and have been characterized functionally, both when reconstituted into artificial lipid bilayers^2^,^20^,^25^ and when inserted into the membranes of cells^26^. Computational studies have been used to explore, for example, their conformational dynamics in relation to ion permeation^27^,^28^. Electroporation has also been used to introduce DNA nanostructures into cells^29^.

A central challenge in the construction of DNTs is, however, how to embed a polyanionic DNA assembly within the hydrophobic environment presented by the core of a lipid bilayer. This hurdle can be overcome by chemically modifying the outer surface of DNT with a band of hydrophobic groups to match the nature of the bilayer core. Successfully tested hydrophobic groups are ethyl groups on phosphate analogues of the DNA backbone^20^ that modifies the DNT surface with a hydrophobic ring and neutralizes the negative charge (see Fig. 1a). A number of variations upon this design has been explored, including moving the hydrophobic band closer to one end of the DNT^26^, or alternatively by replacing the hydrophobic ring by porphyrin^30^ or cholesterol^2^,^18^ tags to anchor the DNT in a membrane. However, the latter modifications do not provide extensive hydrophobic coverage of the DNT surface, and may result in a greater degree of mismatch between the lipid tails which form the bilayer core and the DNT surface. Also, in contrast to the ethyl modifications, the number of cholesterols or porphyrins employed is much smaller, and, thus, the residual negative charge on the DNA surface exposed to the hydrophobic core remains high.

Despite experimental progress in anchoring DNTs into membranes, the molecular structure, dynamics and energetics of the process are unknown. In particular, the molecular structure of the interface between the DNA tube and the lipid bilayer remains unclear. Similarly, the dynamic structural changes of the inserted pore and the energetics of membrane insertion are unknown.

Molecular dynamics (MD) is a powerful tool to explore the interactions of membrane proteins with lipid bilayers^31^,^32^. MD simulations have also been employed to examine the conformational dynamics and permeation properties of nanopores (for example, refs 33, 34, 35, 36, 37, 38, 39, 40, 41), including those formed by non-membrane-spanning DNTs^27^,^42^ and a single bilayer-spanning DNA duplex^43^. Recently, MD simulations have provided an estimate of the free energy substantive barrier to insertion of unmodified DNA into a lipid bilayer, while suggesting that the presence of divalent cations may lower the energetic barrier^44^. The use of coarse-grained (CG) MD is well suited to extend simulation timescales^45^,^46^ thus enabling exploration of the energetics of protein/lipid interactions^47^.

Here, we use MD simulations to explain the interactions of a simple DNA nanopore composed of six interconnected duplexes with a lipid bilayer. Our multiscale MD approach enables detailed exploration of a membrane-embedded DNA nanopore in a lipid bilayer. CG simulations elucidate the stability and interactions of the DNT with the bilayer, while atomistic (AT) simulations provide a detailed picture both of DNT/lipid interactions and of structural dynamics of nanopores. Our simulations reveal that the hydrophobic band on the surface of the nanopore resists extraction of the DNT from the bilayer. The resultant nanopore exhibits selectivity and gating properties that depend on the ionic strength of the surrounding aqueous solution.

## Results

### DNA nanopores in a bilayer

**Figure 1:**
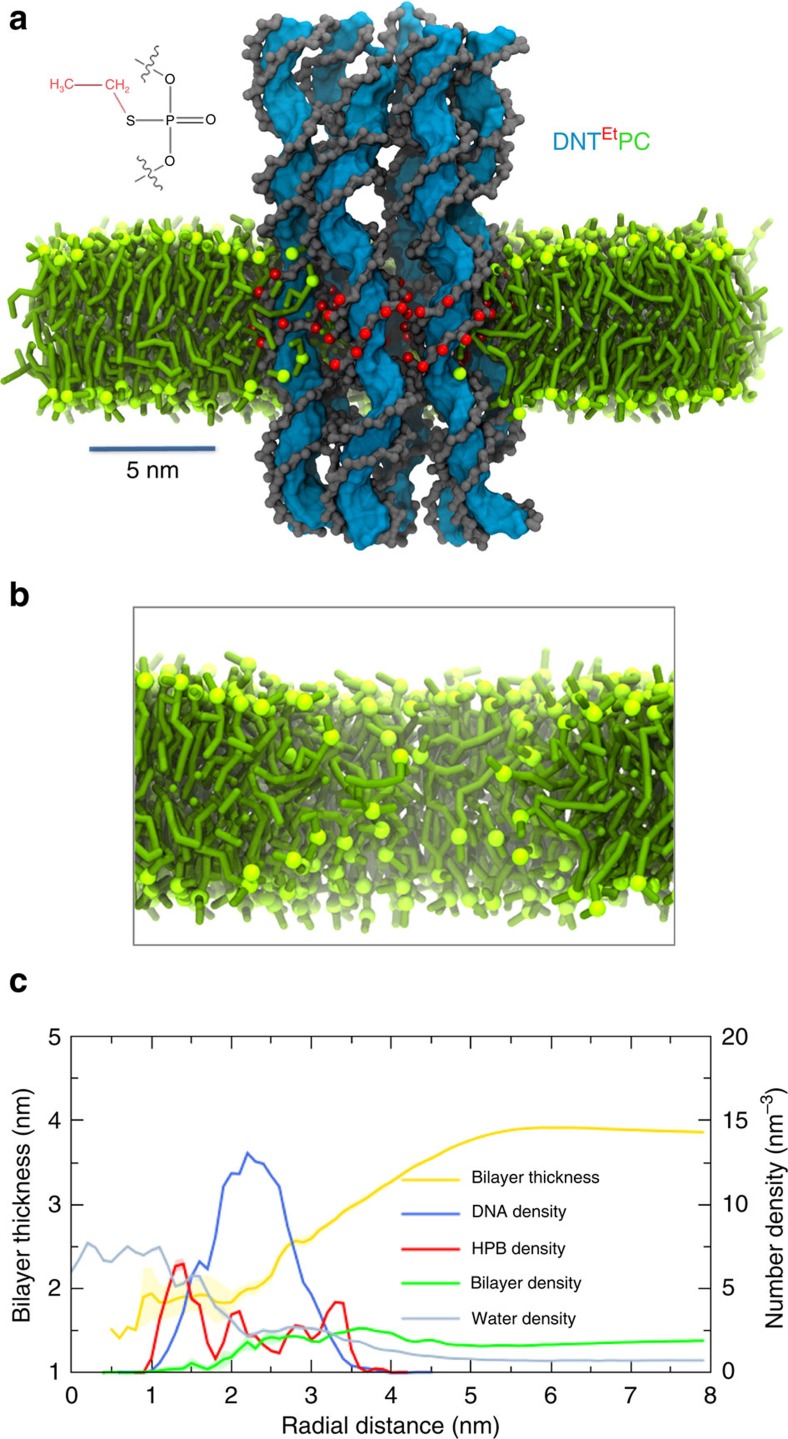
DNA nanopore in a bilayer. (**a**) Snapshot from a CG simulation (DNT^Et^PC; see Table 1 for details and abbreviations) of a simple DNA nanopore composed of six interconnected duplexes, embedded in a lipid bilayer; water and ions are omitted for clarity. The colour scheme is: blue surface=DNA bases; grey surface=DNA backbone; green=PC tail/phosphate head group; red=hydrophobic beads (HPB). (**b**) A zoomed in view of the same system, showing only the lipids and revealing the local perturbation of the bilayer by the inserted DNA nanopore. (**c**) The bilayer thickness (yellow line; left-hand axis) and number densities of the system components (right axis) are shown as a function of radial distance from the centre of the nanopore. Bilayer thickness were calculated as headgroup phosphate–phosphate distances. For number density analysis cylindrical grid shells (0.1 nm thick and 4 nm long, and thus approximating the membrane thickness), were constructed around the central axis of the transmembrane domain of DNT. Thickness and density profiles were averaged over the last 400 ns of each simulation. The results shown are combined and averaged over six different runs. Note that the densities of the lipid bilayer and of the hydrophobic Et (ethyl) groups have been scaled up 5 × for ease of visualization.

CG MD simulations were performed using the MARTINI forcefield, which has proven suitable to probe interactions of a large variety of biomolecules and nanoparticles with membranes^48^. These simulations yielded a model of the DNA nanopore with a hydrophobic surface band (DNT^Et^—see Methods and Supplementary Fig. 1) embedded in a simple model (phosphatidylcholine; PC) lipid bilayer. The starting model for most of the simulations was obtained by coarse-graining a representative structure (obtained by clustering) from a previous simulation of a DNT scaffold in aqueous solution^27^, which was then modified to add a hydrophobic surface band^20^, and inserted in a PC bilayer (see Methods for details). The resultant DNT-bilayer system, with water and ions (1 M NaCl) on either face of the membrane, was simulated for 1 μs (six repeats; see Table 1). A snapshot of the final configuration from one of these simulations is shown in Fig. 1a. These simulations started in most cases with a model in which the DNT was manually inserted so to span a lipid bilayer, or in a test case in which a lipid bilayer was self-assembled about a DNT (both protocols may be used to study the bilayer interactions of, for example, integral membrane proteins^49^). The DNT can be seen to stably span the membrane, with the ethyl groups of the hydrophobic band located within the hydrophobic core of the bilayer. If one examines the bilayer with the DNT removed (Fig. 1b) it can be seen that there is a degree of local reorganization of the lipids in the vicinity of the inserted nanopore, such that the lipid headgroups are drawn into the bilayer core, so that they may form close contacts to the modified DNA.

A more quantitative view was obtained by examining the bilayer thickness and the distribution of different components as a function of the radial distance from the centre of the nanopore (Fig. 1c). The thickness profile reveals that membrane thinning occurs around the DNT and persists for up to *∼*2 nm away from its surface. As in our previous (AT) simulations of a DNT in aqueous solution^27^, water is present both within the lumen of the nanotube and also (to a lesser extent) within the walls of the nanotube itself. Lipid density can be seen to overlap radially with DNA density, confirming the close interactions of lipid molecules and DNA of the nanopore noted above. The lipid density can also be seen to overlap with the radial density of the ethyl groups of the hydrophobic band (which are present on both the inner and outer surfaces of the nanotube as well as within the walls of the nanotube). Lipid molecules are absent from the lumen of the nanotube and so do not ‘clog' the pore.

Simulations were performed to assess the robustness of the results to variations in the structure of the nanotube, the nature of the bilayer and the simulation conditions employed (Table 1 and Supplementary Figs 2 and 3). Thus, in simulation iDNT^Et^PC an initial DNT model which had not been subjected to AT simulations in aqueous solution prior to bilayer insertion was employed, whereas in DNT^Et^PCsoft a ‘softer' (that is, lower force constants—see Methods) elastic network model (ENM) was used to maintain the DNA conformation. These ‘softer' and ‘harder' models may be thought of as possible limiting cases for the degree of dynamic flexibility seen in AT simulations (see below). As might be anticipated, the softer ENM results in some smoothing of the density profiles while the iDNT model results in sharpening of the density profile peaks (relative to the DNT^Et^PC simulations), but the overall distributions of components and the bilayer thickness profile are not substantially changed. Changes in the lipid bilayer composition (the inclusion of 20% of the anionic headgroup lipid PS) or to the lipid parameters (iDNT^Et^PCold) or to the degree of hydrophobicity of the nanopore (DNT^6Et^PC) likewise did not result in any substantive changes. We also simulated the nanopore in two more complex lipid bilayers, namely a simplified model of the lipid mixture in a bacterial (*E. coli*) membrane^50^ and a model of a mammalian cell membrane^51^. The bacterial membrane was modelled by a PE/PG (3:1) bilayer and the mammalian cell membrane by an asymmetric bilayer with an outer leaflet containing sphingolipids and glycolipids and an inner leaflet containing negatively charged PS and PIP_2_ lipid molecules. The results (shown in Supplementary Fig. 3) indicate that the local thinning of the bilayer is also seen in the more complex mixed lipid membranes. The mammalian membrane simulations suggest a small degree of depletion of anionic lipids (PS and the ganglioside GM3) adjacent to the inserted nanotube, as might be anticipated. However, this differences from the nanotube in a zwitterionic PC bilayer are relatively small. Thus we may conclude that the overall simulation results are robust to minor changes in the details of the simulation model and the parameters, as well as lipid composition.

We wished to determine whether a substantially smaller hydrophobic band altered the behaviour of the nanopore within the membrane. To this end we simulated the behaviour of three systems with either a single ring of ethyl groups around the nanopore (DNT^1Et^PC) or with just two such rings (DNT^2AEt^PC and DNT^2DEt^PC; see Supplementary Fig. 1 and Table 1 for details). The overall behaviour of each of these nanopores was similar to that of the nanopores with either 6 or 7 rings of ethyl groups (see above). However, analysis of the distribution of tilt angles (between the long axis of the DNT and the bilayer normal; see Supplementary Fig. 4) revealed that nanotubes with fewer rings of ethyl groups ‘wobbled' more in the bilayer, as evident from a shift in and broadening of their tilt angle distributions. This is likely to be an important consideration when designing hydrophobically derivatized DNTs, as a wide range of tilt angles might be anticipated to perturb ion flow through the nanopores. Thus, it has been suggested that lipids may be able to occlude nanotubes^52^, and this would be expected to be more likely to occur if nanopores were prone to tilting, especially if shorter (relative to the membrane thickness) DNTs were to be explored^2^.

### Local lipid interactions

**Figure 2:**
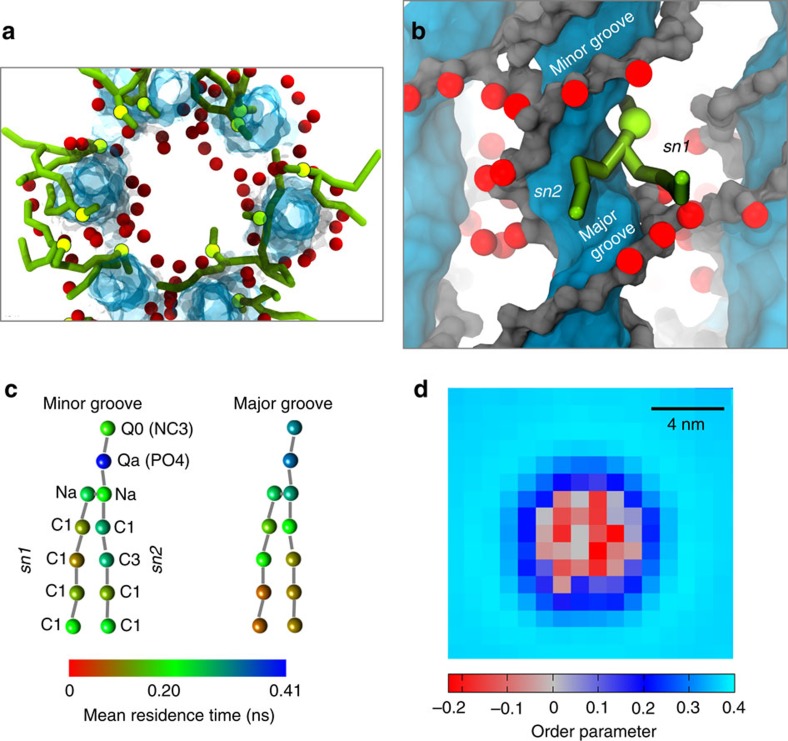
Local lipid interactions. (**a**) Snapshot from the CG simulation (DNT^Et^PC; see Table 1 for abbreviations) showing presence of locally perturbed lipids (green) around the inner surface of the DNT (blue/grey with Et groups in red) lumen. Lipids do not plug the lumen, but some are exposed on the luminal surface. (**b**) Snapshot of the same system showing a selected lipid, the headgroup and *sn2* acyl chain of which interact with the major groove surface of the DNA. (**c**) Patterns of interaction of lipid CG particles (coloured as mean residence times) with minor and major grooves of DNT^Et^. Residence times for lipid/DNA contacts were calculated for each bead of perturbed lipids only, using a cutoff distance of 0.5 nm for contacts. Labels along each bead represent bead type, and labels in parenthesis show bead name for CG MARTINI. See Supplementary Fig. 5. (**d**) Order parameter of lipids sampled in square grids (1 × 1 nm^2^) projected in *xy* plane and centred around DNT^Et^ at (0, 0). Lipids *xy* positions refer to the phosphates of the lipid headgroups. Lipid order parameter was calculated for all the lipids in each grid box and averaged over all simulation frames for each run, and then for all six runs. The order parameter was calculated using: 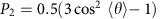.

Next we explored the interactions of lipids adjacent to the membrane-spanning DNT (that is, those next to the TM domain) in more detail. Visualization (Fig. 2a) revealed that a number of lipids penetrated the DNT to the extent that their head groups pointed into the lumen of the nanopore. The tails of the lipid molecules in contact with the DNT could be seen to wrap around the DNA double helix, in both the major and (to a lesser extent) the minor grooves (Fig. 2b). Such locally perturbed lipids remain in close proximity to the DNT along the width of the hydrophobic band. We examined the pattern of residence times (see also Supplementary Fig. 5) in the minor and major grooves for the different constituent particles of the CG lipid molecules. For both the DNT^Et^PC (Fig. 2c) and the iDNT^Et^PC (data not shown) simulations the *sn2* chain interacts with both the major and minor grooves of the DNA more than does the *sn1* chain. The choline and phosphate groups (NC3 and PO4 particles; Fig. 2c) also exhibited preferential interactions with the major groove. This analysis along with the observed local thinning of the bilayer (see above) reveals that the interactions of lipid molecules with the surface of the DNT induce a local perturbation of the lipid bilayer structure. This can also be seen in the order parameters for the lipid tails in close proximity to the surface of the DNT (Fig. 2d).

### Metastable and stable states

**Figure 3:**
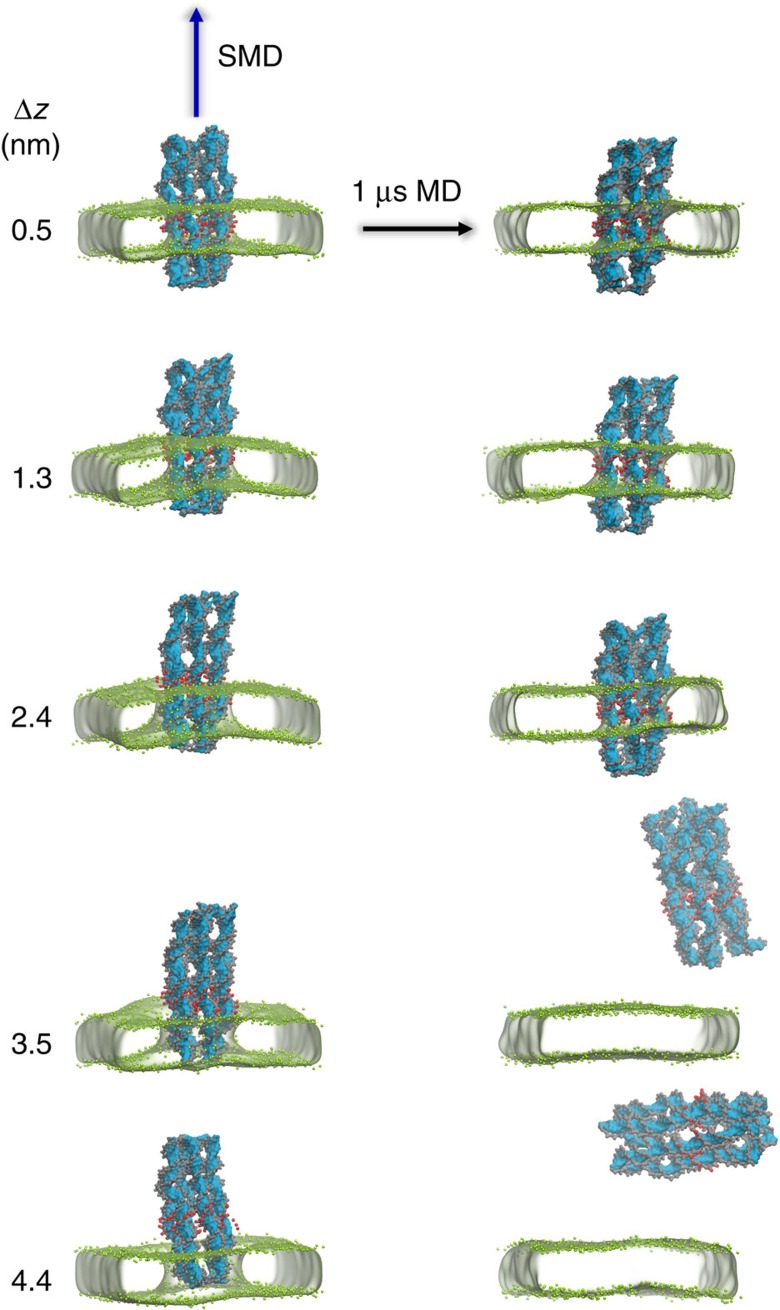
Displacing the DNA nanopore relative to the bilayer. Five displaced states were generated by using SMD to displace DNT^Et^ (see Table 1 for abbreviations) by Δ*z* along the bilayer normal relative to its equilibrium position in the membrane. The left-hand column shows images of the five initial-displaced structures (DNT in blue/grey/red; PC bilayer as a surface defined by the green phosphate particles). Each displaced structure was used as the starting point for an unrestrained 1 μs simulation, yielding the final structure snapshots shown in the right-hand column. Note that for Δ*z*=0.5 and 1.3 nm, the red Et particles are initially within the bilayer, for Δ*z*=2.4 nm about half of the Et particles are within the bilayer, whereas for Δ*z*=3.5 and 4.4 nm the particles are outside of the bilayer.

**Figure 4:**
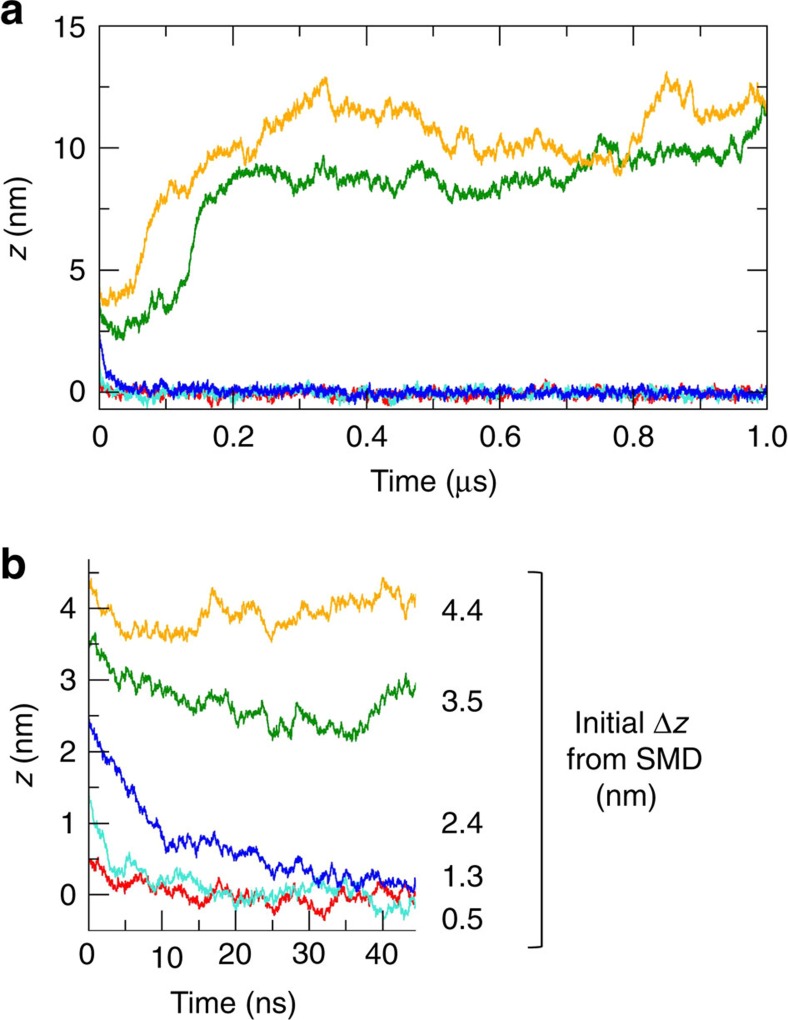
Response to displacement. (**a**) Position along *z* (the bilayer normal) of the centre of mass (COM) of the DNT hydrophobic region relative to the COM of the bilayer as a function of time for the five displaced simulations shown in Fig. 3. (**b**) An expanded version of the same graph corresponding to first 45 ns of each simulation. The colour code is: Δ*z*=0.5 (red), 1.3 (cyan), 2.4 (blue), 3.4 (green) and 4.4 (yellow) nm.

To explore further the (meta) stability of the DNT when embedded in a lipid bilayer, we used steered MD (SMD)^53^ to perturb the system generated at the end of the 1 μs DNT^Et^PC simulation by displacing the DNT relative to the bilayer (Fig. 3). The DNT was displaced to differing extents ranging from Δ*z*=0.5 nm to Δ*z*=4.4 nm (Δ*z* measured as distance between the centres of mass (COM) of bilayer and hydrophobic band). Each perturbed system was then used as the starting point for a further unrestrained 1 μs simulation to examine whether or not the DNT relaxed back to its initial position in the bilayer. Note that for Δ*z*=0.5 and 1.3 nm, the hydrophobic bands (that is, the CG ethyl particles) are initially within the bilayer, for Δ*z*=2.4 nm only about half of the ethyl groups are within the bilayer, and for Δ*z*=3.5 and 4.4 nm the hydrophobic groups are outside of the bilayer. The behaviour in these five simulations can be measured by tracking the COM of the DNT relative to that of the bilayer (Fig. 4). In the case of the smaller displacements (Δ*z*=0.5, 1.3 and 2.4 nm) the DNT relaxes back to its initial position within ∼40 ns. In contrast, for the larger displacements (Δ*z*=3.5 and 4.4 nm) which completely removed the hydrophobic band from the bilayer core, the DNT exits from and diffuses away from the bilayer into the bulk solvent. This suggests that there are two (meta)stable states of the system, one with the DNT spanning the membrane and one with the DNT free of the membrane.

**Figure 5:**
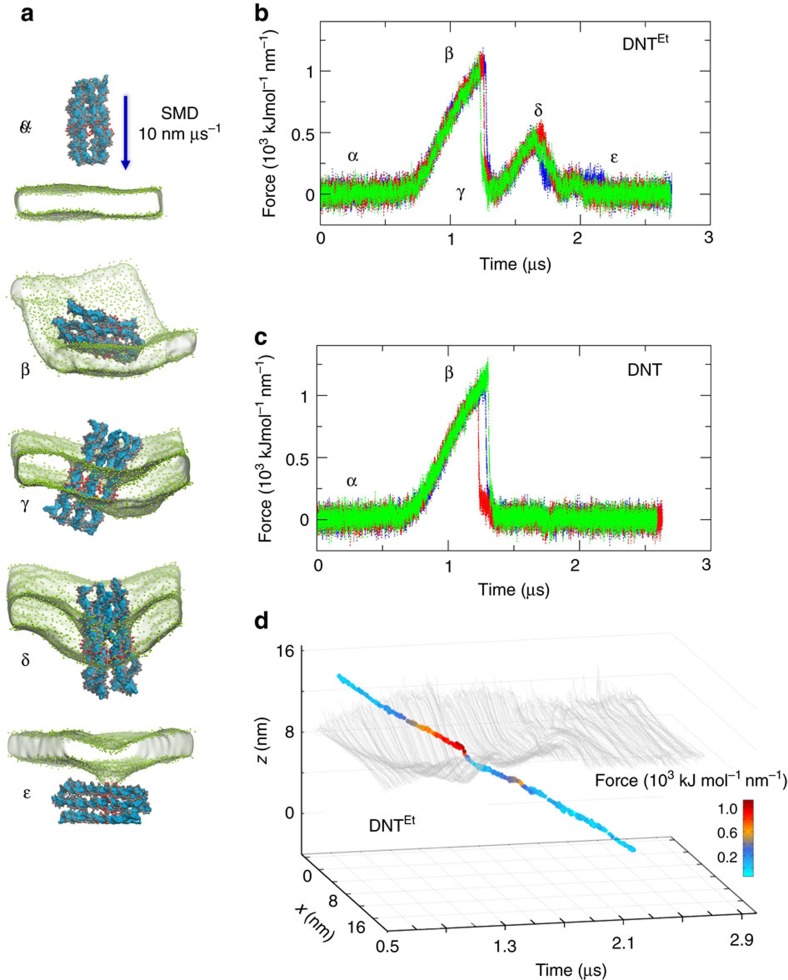
Pushing DNA nanopores through a bilayer. (**a**) Snapshots α to ɛ from a SMD simulation in which DNT^Et^ (see Table 1 for abbreviations) was moved at a rate of 10 nm μs^−1^ along the normal to a PC bilayer. In the snapshots shown the nanopore approaches the bilayer (α), interacts with the surface of the bilayer (β), penetrates and spans the bilayer (γ), is pulled out of the bilayer (δ) and finally exits the bilayer (ɛ). (**b**) A plot of force versus time for the SMD simulations of DNT^Et^ illustrated in (**a**). Three repeat simulations are shown in three different colours. (**c**) A comparable plot for SMD simulations of the unmodified (that is, no Et groups) DNA nanopore. (**d**) A multi-dimensional plot showing the time evolution, as 4 ns time steps, for the DNT^Et^ simulation in (**a**), showing membrane surface distortion (grey lines; calculated as the *xz* projected COM of the PC beads), the *xz* projected coordinate of the COM of the Et modified region of the DNT versus time, with the points coloured by the corresponding force exerted. This analysis is also shown as a movie in the Supplementary Movie 1.

To explore the nature of the barrier separating these two states we performed further SMD simulations, comparing the DNA nanopore both with (DNT^Et^PC) and without (DNT PC) the hydrophobic band on its surface (see Table 1). In these simulations, the DNT was initially located outside the bilayer, and then was pushed into and across the bilayer at a constant velocity of 10 nm μs^−1^ (Fig. 5a). It can be seen that the DNT experiences some resistance (as evident from the bilayer curvature) as it enters the bilayer, then adopts a transbilayer configuration and then experiences further resistance as it exits from the bilayer. The force profile (Fig. 5b) is remarkably consistent between the 3 repeat simulations and shows a gradual build up of force to ∼1,000 kJ mol^−1^ nm^−1^ (equivalent to a single-molecule force of ∼1,700 pN) as the DNT is pushed into the membrane. Note that this force is approximately the same whether or not the DNT has a hydrophobic surface band (Fig. 5b,c). It can be seen that in resisting entry to the bilayer the DNT twists away from the bilayer normal. If the DNT is restrained so that its long axis is parallel to the bilayer normal (that is, pushing it into the bilayer like a nanoneedle^54^; Supplementary Fig. 6A) the force profile is similar but the peak force on entry is reduced to ∼800 kJ mol^−1^ nm^−1^). Once inserted into the bilayer the force drops to zero. Then as the DNT is pushed through the bilayer and starts to exit, if there is a hydrophobic band then there is a further barrier. However, if the DNT does not have a hydrophobic band this barrier is absent and the DNT can be pushed right out of the bilayer without any resisting force. If the hydrophobic band is present, the barrier for exit from the bilayer is ∼400 kJ mol^−1^ nm^−1^. We also observe that this barrier is dependent on the thickness of the hydrophobic band (see Supplementary Fig. 6B,C). In general, it can be concluded that for the same DNT with different hydrophobicity levels, the insertion barrier remains about the same, but that the exit barrier, which would determine the stability of the inserted complex, is reduced as the thickness of the hydrophobic band is reduced. Furthermore, if one starts with a DNT pre-inserted into a bilayer and SMD is used to pull the DNT out of the bilayer, there is again a barrier of 400 to 500 kJ mol^−1^ nm^−1^ for exit from the bilayer (see Supplementary Methods and Supplementary Fig. 7). The forces experienced on entry into and exit from the bilayer of the DNT are mirrored by local perturbation of the bilayer curvature (Fig. 5d), the bilayer relaxing back to a plane once the DNT is inserted in a transmembrane orientation, and once again when the DNT has exited.

Although simulations of pushing a DNT through a bilayer may seem to be rather artificial (as the rate of translation is much higher than that which might be achieved experimentally) it provides a computational tool which allows us to probe the free energy landscape for DNT/bilayer interactions in terms of possible barriers for entry into the bilayer from the aqueous phase and for exit from the bilayer once inserted. The perhaps surprising result is that the hydrophobic ethyl group band on the surface of the nanotube stabilized it within the membrane not by facilitating entry but by providing a barrier to exit, that is, once in the membrane the nanotube is less likely to leave if the hydrophobic band is present on its surface. This is an important principle for future DNT designs.

One may compare the peak force for exit of the DNT^Et^ from the bilayer (∼500 kJ mol^−1^ nm^−1^, equivalent to ∼800 pN) with (experimental) estimates of up to ∼300 pN for AFM unfolding and extraction of membrane proteins from lipids bilayers^55^,^56^. However, it is important to note that there are several orders of magnitude of difference in the pulling rate in SMD and in the AFM experiments, and indeed only by extrapolation to zero pulling rates (with an exponential dependence of force of pulling speed) may equilibrium estimates of free energy landscapes be obtained^57^.

### AT simulations

Having used CG simulations to develop and characterize a model of the DNT^6Et^ in a lipid bilayer, we backmapped the final CG configurations^58^ to enable more detailed AT simulations in order to explore the conformational dynamics of the nanopore while within a lipid bilayer environment. We simulated a membrane inserted nanopore (DNT^6Et^) with two different ionic strengths in the aqueous phase: 0.15 M NaCl (corresponding approximately to normal physiological saline, and so facilitating comparison with experiments on nanopores inserted into cell membranes^26^), and 1.0 M NaCl (as in the CG simulations), enabling comparison with *in vitro* experiments on ionic currents through nanopores in artificial membranes^20^. Each simulation, containing ∼0.83 × 10^6^ atoms, was run for 0.5 μs (see Table 1).

**Figure 6:**
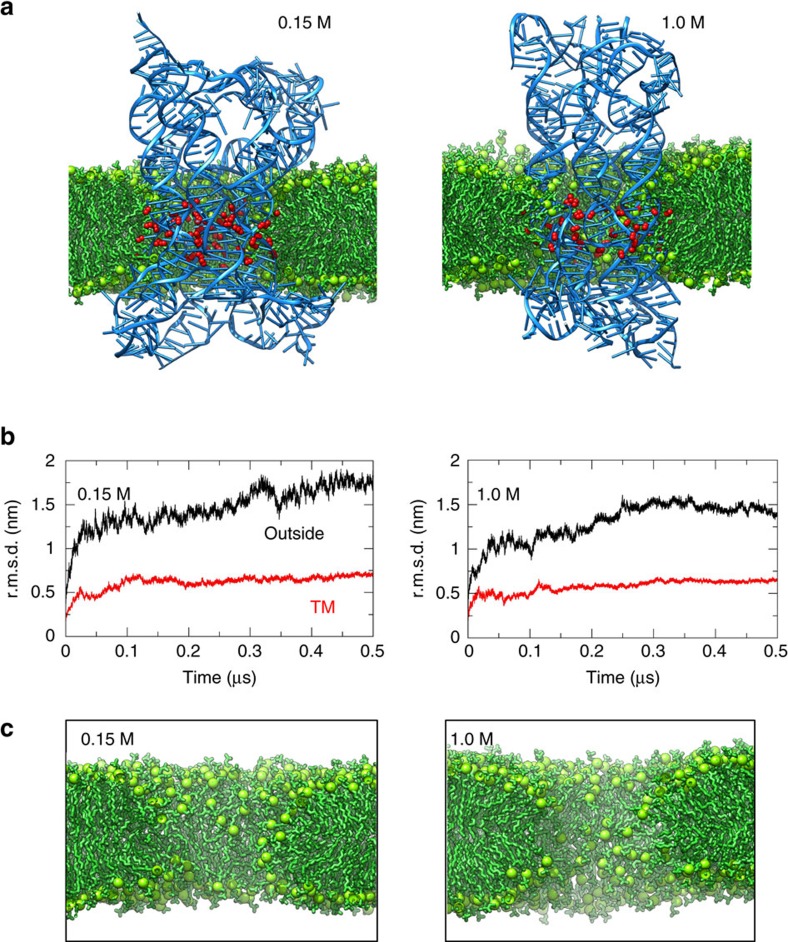
AT simulations of a DNA nanopore embedded in a POPC bilayer. Snapshots and graphs on the left- and right-hand sides are for simulations in 0.15 M and 1.0 M NaCl, respectively. (**a**) Final (0.5 μs) snapshots for simulation AT-DNT^6Et^; see Table 1 for abbreviations (green sticks and balls=lipid tails and head groups, respectively; blue ribbon ladders=DNA; and red beads=ethyl chains; water and ions omitted for clarity). (**b**) r.m.s.d. for the DNA heavy (that is, not H) atoms relative to the starting structure as a function of time. r.m.s.d. graphs are shown separately for the transmembrane (TM; red) and outside-the-membrane (black) regions of the DNA. (**c**) A zoomed in view of the same systems, showing only the lipids and revealing the local perturbation of the bilayer by insertion of the DNA nanopore. Compare with Fig. 1b.

It can be seen that at 0.15 M the DNT undergoes a substantive change in conformation from the starting structure compared to the simulation at 1.0 M (Fig. 6a; also see Supplementary Fig. 8). This comparison can be quantified by measuring the root mean square displacement (r.m.s.d.) of the DNA heavy (that is, not H) atoms relative to the starting structure as a function of time (Fig. 6b). For both the TM and the outside-the-membrane regions of the DNA, as expected the r.m.s.d. shows an initial jump as the structure from the (ENM restrained) CG simulations relaxes. At both ionic strengths the r.m.s.d. is then approximately constant for the TM region of the nanopore, indicative of its conformational stability in the membrane environment. The r.m.s.d. is higher for the outside region indicative of greater structural deformation, especially so in the 0.15 M simulation. The greater conformational stability of the outside region in the presence of 1.0 M NaCl suggests that the increased ionic strength helps to stabilize the densely packed DNA-origami structures. Published studies of a similar nanopore in 1.0 M KCl (ref. 28) showed a comparable degree of conformational flexibility, as is also observed for the un-derivatized DNT in the absence of a membrane^27^. The lipid organization around the nanopore in the AT simulations (Fig. 6c) remains similar to that observed at the CG level, with no apparent effect of the salt concentration in line with the similar r.m.s.d. of the TM parts. More detailed examination of the simulation (sample snapshots are shown in Supplementary Fig. 8B) shows that lipid tails remain within the major and minor grooves of the membrane-spanning DNA segments.

**Figure 7:**
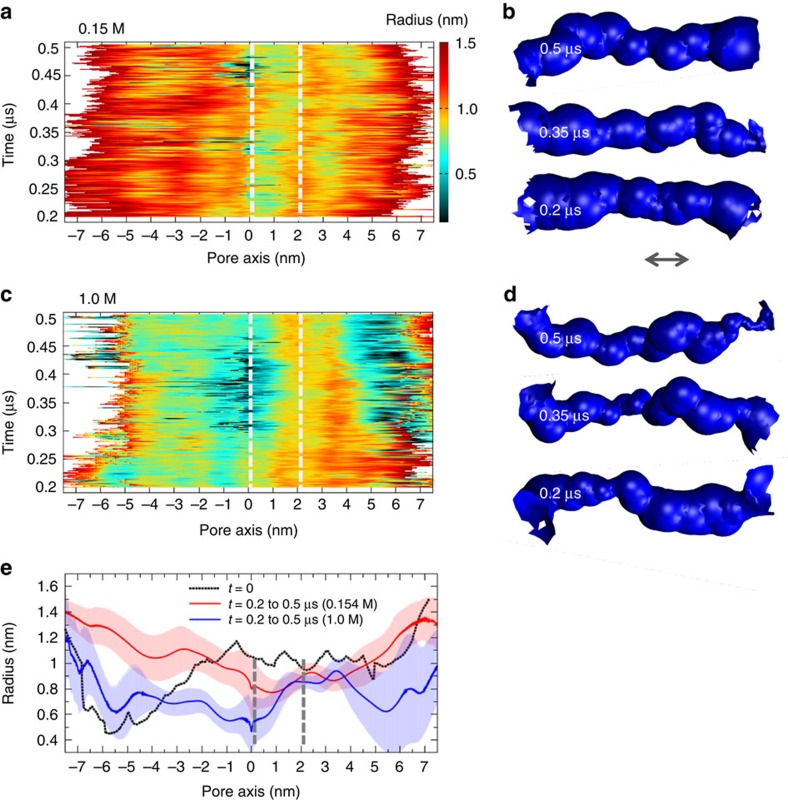
Pore dynamics from the AT simulations. (**a**,**c**) The pore radius profiles (calculated using HOLE^75^) of the DNT radius is shown as a function of time for the simulations in (**a**) 0.15 M and (**c**) 1.0 M NaCl. Vertical-dashed white lines represent the approximate location of the bilayer projected onto the pore axis. This was obtained by juxtaposing radius profiles for each frame after 20 ps. (**b**,**d**) Snapshots of the pore-lining surfaces in the transmembrane region (indicated by the arrow) of the nanopores at 0.2, 0.35 and 0.5 μs within each simulation. (**e**) Time-averaged pore radius profiles for the simulations in (red) 0.15 M and (blue) 1.0 M NaCl. The error bars correspond to one standard deviation either side of the mean. The pore radius profile of the initial structure is shown in black.

In our previous simulations of the un-derivatized DNT in aqueous solution, we observed time-dependent fluctuations in the pore radius, which we suggested might provide a model for pore gating^27^. We therefore explored the effect of the bilayer and the ionic strength on fluctuations in the radius of the transmembrane pore by evaluating the pore radius profile as a function of time for each of the simulations (Fig. 7a–d). At the lower ionic strength (0.15 M) the radius profile suggests that in the external regions the pore lumen remains open (radius ∼1 nm) for most of the time; the TM region is a little narrower at radius ∼0.8 nm but also remains open throughout the simulation. At the higher ionic strength (1.0 M) the fluctuations in the pore radius are more pronounced and overall there is a degree of contraction of the pore relative to the starting structure (Fig. 7e). Thus the time dependent radius profile reveals opening and closing events (‘gating' motions) both close to the ‘upper' mouth of the TM pore (that is, at z∼0 nm in Fig. 7c,d) and in the ‘lower' outside-the-membrane region (z∼+5 nm). These are similar to the pore radius fluctuations seen in our previous study of DNT simulations in 1.0 M KCl aqueous solution^27^. These differences between the behaviour of the pore at the two ionic strengths are reflected in their time-averaged pore radius profiles (Fig. 7e). In each simulation the time-averaged distributions of ions (Supplementary Fig. 9) indicate that the pore is cation selective. At 0.15 M the ratio of Na^+^ to Cl^−^ in the lumen of the pore is ∼14; at the higher ionic strength it is ∼2. Although some hydrophobic ethyl groups are located in the luminal lining of the pore this is not sufficient to cause hydrophobic gating^3^ and so fully hydrated ions and water molecules remain within the lumen of the nanopore throughout the simulations. Thus, in summary, in terms of pore properties at lower ionic strength the DNA nanopore has a high cation selectivity and a higher probability of being open.

**Figure 8:**
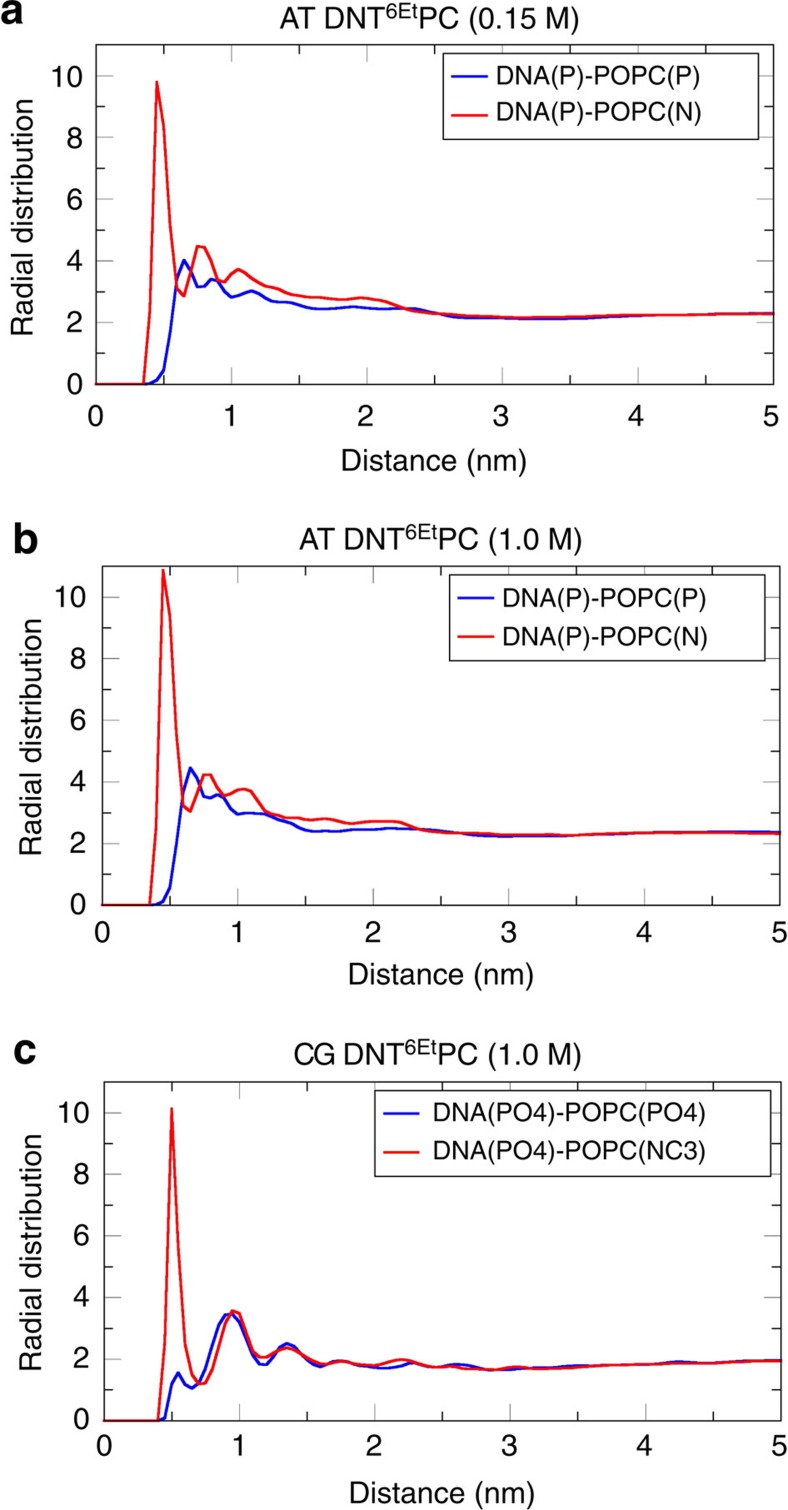
Radial distributions of lipids around the DNA nanopores embedded within the bilayer. (**a**,**b**) Radial distributions functions (RDFs) for DNA-phosphorus to PC headgroup phosphorus (blue), and DNA-phosphorus to PC headgroup (choline) nitrogen (red) for the AT-DNT^6Et^ (see Table 1 for abbreviations) simulations in 0.15 and 1.0 M NaCl, respectively. (**c**) The equivalent radial distribution functions for the CG DNT^6Et^ simulation, for DNA-phosphate particles to PC headgroup phosphates (blue), and DNA-phosphates to PC headgroup choline particles (red). Only negatively charged (that is, not ethylated) DNA bases near the membrane surface were included in this analysis in both the cases.

Turning to the lipids, the radial densities (Supplementary Fig. 9A) are comparable to those from the CG simulations, and confirming a degree of penetration of the ‘walls' of the DNT by lipid molecules (seen as overlap of lipid and DNA radial density). However, the lipids do not clog the pore lumen. The AT simulations also provide an opportunity to explore in more detail the interactions of lipid molecules with the inserted DNT. Examination of radial distribution functions (Fig. 8a–c) provides clear evidence for favourable interactions of the anionic phosphates of the DNA backbone and the positively charged choline groups of the zwitterionic lipid headgroup. Thus, local reorganization of the lipids occurs so as to stabilize the DNA nanopore within the bilayer core.

Inspection of the distributions of ions and of water around the inserted nanotube (Supplementary Fig. 9B) suggests that there is not a substantial number of waters trapped between the nanopore and the surrounding lipid molecules. Therefore, there is unlikely to be significant ion ‘leakage' possible around the edges of the pore. It is interesting to compare this with a recent study of a large conductance DNA ‘porin'^59^ for which simulations suggest ca. 20% of the ionic current may flow around the outside of the channel.

Current leakage around or through outside the nanotube wall might be anticipated due to the porous^27^ nature of DNA nanopore. However, this porous nature would be expected to be suppressed by surrounding lipids once the nanopore is inserted in a bilayer. However, as noted above we did observe a degree of local membrane thinning (Fig. 1c) around the DNT. This prompted us to examine test a case in which the hydrophobic patch on the DNT was extended beyond the membrane thickness (DNT^nEt^; see Table 1). For this system we observed (Supplementary Fig. 10) a smaller degree of membrane thinning than was seen around DNT^6Et^. Thus by extending the hydrophobic surface coating possible current leakage outside the DNA walls may be reduced.

### Comparison with experiment

**Figure 9:**
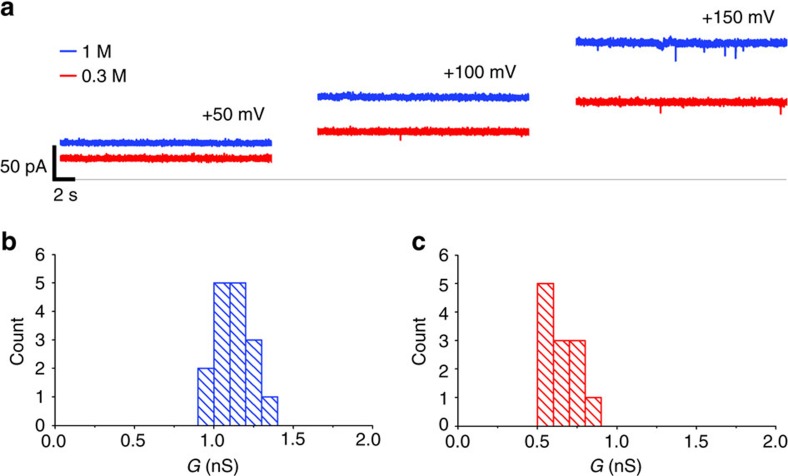
Ionic currents through DNA nanopores. Nanopores were inserted in diphytanoyl PC bilayers and single-pore currents were measured in the presence of 0.3 M (red) and 1.0 M (blue) KCl (see Supplementary Methods for details). (**a**) Representative ionic currents measured in response to successive voltage steps of +50, +100 and +150 mV. (**b**,**c**) Conductance histograms derived from pore current recordings in the presence of (**b**, blue) 1.0 M and (**c**, red) 0.3 M KCl.

From the AT simulations described above we predict that as a result of reduced electrostatic screening at a reduced ionic strength (0.15 M in the simulations) the DNA nanopore is expected to be somewhat wider and to have a higher probability of being open than under ‘standard' (1.0 M salt) conditions. It should be noted that the simulation measurements of pore properties are resolved on a sub-microsecond timescale. On an experimental timescale (seconds) we would expect these two effects (that is, a wider pore and a higher open probability) to be combined into a higher effective conductance at the lower ionic strength than would otherwise be the case due simply to a difference in ionic concentration. We tested this prediction by measurement of channels currents at a low (0.3 M) and high (1.0 M) salt concentrations (see Fig. 9, Supplementary Methods Table 1 and Supplementary Figs 11 and 12 for details).

From experimental measurements (Fig. 9), the nanopore conductance is *g=*0.6 and 1.1 nS in the presence of 0.3 and 1.0 M KCl, respectively. Thus *g*_1.0M_*/g*_0.3M_=1.8, which is substantially smaller than the value of 3.3 which would be expected on the basis of the ionic concentration difference alone. We can compare simple predictions of the conductance based on the expression for pore conductance (given in ref. 60 and discussed further in ref. 61) *g*_PORE_=π*R*^*2*^*/ρL* for a pore of radius *R*, length *L*, and filled with an electrolyte solution of resistivity *ρ*. Using the time-averaged radius profiles from the simulations (Fig. 7e) we estimated the ratio of *g*_1.0M_*/g*_0.3M_*=*2.3 (assuming that the profile obtained from the 0.15 M simulation approximates that at 0.3 M as both are ‘low' salt concentrations). Thus we can conclude that the prediction of a lower measured conductance at higher ionic strengths due to narrowing of the pore and decrease in the opening probability combined is indeed observed experimentally, providing strong support for our simulations.

We also measured the melting temperature of the DNT (in small unilamellar vesicles) in the presence of 0.3 M (52 °C) and 1.0 M (63 °C) KCl. Thus, the DNT appears to be less stable at the lower ionic concentration, and so at room temperature might be anticipated to exhibit greater flexibility than at 1.0 M. This correlates with the higher r.m.s.d. for the extra-membraneous region of the DNT at low ionic concentration relative to at 1.0 M (Fig. 6b).

## Discussion

We have applied CG followed by AT simulations to explain the origin of the stability of an archetypally simple DNA nanopore spanning a phospholipid bilayer. Although the overall DNT carries a high negative charge density, the central hydrophobic band stabilizes the DNT in a bilayer by providing an energetic barrier to exit of the inserted nanopore. This occurs alongside a local reorganization of the bilayer structure so as to optimize interactions with the nanopore, by lipids interacting with the surface grooves of the DNA. Thus, the conductance properties of these DNA nanopores reflect the interactions of ions and water molecules with the luminal and pore-lining walls of the nanopore, while the stable localization of the DNT within the bilayer is determined by the hydrophobic bands. This demonstration of modularity of pore function and membrane stability will guide design of next-generation DNA nanopores with tailored gating and ion selectivity properties. Such designs will benefit from our understanding of both simple DNA nanopores, as in the current study, and, for example, of more complex DNA origami `porins'^59^.

The nanopore is dynamic, and responsive to both the bilayer^28^ and aqueous environment. Thus, the open state of the central pore is stabilized against fluctuations (leading to partial pore closure) by lower (more physiological) ionic strengths. Although at low ionic strength (0.15 M) there are substantive fluctuations in the conformation of the DNT regions outside of the bilayer, the interactions with the lipids protect the TM pore against collapse, as evidenced by a stable pore radius profile in this region. In contrast, at higher ionic strengths, (1.0 M) the fluctuations in the DNT are overall lower, but more pronounced fluctuations in the pore radius occur. Furthermore, the ion selectivity of the nanopore (as evidenced by the ratio of cations to anions in the pore lumen) shows a dependence on ionic strength.

These insights inform design of the next generation of DNTs to selectively permeabilize cell membranes for potential biotherapeutic applications^26^. In particular, the interplay between the role of the lipids and of the electrolyte in stabilizing an open DNA nanopore within a bilayer offers intriguing possibilities for design of novel environment-sensitive gating of DNTs.

## Methods

### Modelling

The DNT model was generated as described previously^27^. Briefly, an initial model was constructed starting from six B-DNA double helices placed on a hexagonal grid with an inter-helix spacing *d*=2.0 nm. Junctions were introduced by breaking and re-joining the strands as in the experimental design^20^. This scaffold was then used to generate an initial DNT model containing 14 single strands of DNA. This model was equilibrated by AT simulations in aqueous solution (1.0 M KCl) for 0.17 μs and cluster analysis (see Supplementary Methods) was used to select a representative conformation.

A representative AT structure of the DNT was converted to a CG MARTINI model using a modified version of ‘*martinize.py*' for our origami DNT^62^. An ENM was used to restrain the DNA conformation during the CG simulations: an elastic network was created for each of the six double helices as well as between the staples that form the junctions. Stiff ENM was used unless stated. For soft ENM a lower force constant value 100 kJ mol^−1^ nm^−2^ was used. Hydrophobic particles, mimicking ethyl-phosphorothioate, were attached to selected phosphate beads of the DNT to generate hydrophobic surface bands (Fig. 1a and Supplementary Fig. 1).

### CG simulations

The CG DNT^Et^ models were embedded in a phospholipid (POPC) bilayer using the ‘*insane.py*' script^63^. The hydrophobic domain was centred in the hydrophobic core of the membrane. No lipids were inserted inside the DNT lumen. CG water particles were also included using ‘*insane*', and the ionic concentration was set to 1 M NaCl. The final dimensions of the simulation box were *c.a.*18 × 18 × 25 nm^3^.

GROMACS (www.gromacs.org) v4.6.5 (ref. 64) was used for CG simulations with the MARTINI DNA^62^ and lipid^63^ forcefields. To remove the initial close contacts, the solvated DNT-membrane systems was subjected to steepest-descent energy minimization. The minimized system was then equilibrated for 1 ns with a time step of 10 fs. Semi-isotropic pressure at 1 bar was controlled by the Berendsen barostat^65^ with a 3 ps coupling constant. A velocity rescale thermostat^66^ was used to maintain temperature at 300 K with 1 ps coupling constant. A cutoff of 1.2 nm was used for Coulombic interactions, shifted between 0.0 and 1.2 nm, and van der Waals interactions were shifted between 0.9 and 1.2 nm. The neighbour list was updated after every 10 steps with a cutoff distance set to 1.4 nm. These parameters correspond to the ‘common' set of parameters for the MARTINI forcefield^67^. Repeat simulations were run by generating new random seeds for velocities. Production simulations were for up to 1 μs. Details of SMD simulations are provided in the Supplementary Methods.

### AT simulations

A final snapshot of the DNT^6Et^-PC CG simulation was converted to all-atom configuration using *backward.py*^58^. DNA, POPC, water were back mapped to all-atom (CG ions were mapped as all-atom water) but later ions were added adjusted to 0.15 M or 1.0 M NaCl concentration by randomly removing some waters. Ethyl modifications were made to selected residues based on the experimental design positions (Supplementary Fig. 1), using ethyl-phosphates (instead of ethyl-phosphorothioate)^28^. Parameters for the ethyl-phosphate modified nucleotides were derived with CGenFF^68^, and other parameters were from CHARMM36 (ref. 69).

AT MD simulations used GROMACS v5.0.2. The initial system configuration was energy minimized using steepest descents. Velocities were generated with 100 ps NVT simulation at 310 K. During this step positional restraints on DNA and lipids were applied in *xyz* with a force constant of 1,000 kJ mol^−1^ nm^−2^. A subsequent NPT simulation was run and positional restraints were removed slowly over ∼2 ns. An unrestrained NPT simulation of up to 0.5 μs was then run. Time steps of 1 and 2 fs was used in the NVT and NPT simulations, respectively. Bond lengths were were constrained using the LINCS algorithm^70^ . A velocity rescale thermostat^66^ was used to maintain temperature at 310 K with a 0.1 ps coupling time constant. The Parrinello–Rahman algorithm^71^ was used to maintain semi-isotropic pressure at 1 bar with a 1 ps time constant. The neighbour list was updated after every 10 steps with a 1.2 nm cutoff. For Coulombic interactions PME^72^ was used with the real space cutoff set to 1.2 nm, while the van der Waals interactions were switched to zero between 0.8 and 1.2 nm.

### Analyses

Locally written PERL scripts, and GROMACS utilities were used for analyses. VMD^73^ and CHIMERA^74^ were used to generate the images. DNT radius profiles were analysed using HOLE^75^. Unless otherwise stated, for CG simulations all the analyses were performed on the last 400 ns of the trajectory (saved every 50 ps) and averaged over repeats of simulations, and for AT simulations all the analyses were performed on the last 300 ns of the trajectory (saved each 20 ps).

### Data availability

Data are available on request to the corresponding authors.

## Additional information

**How to cite this article:** Maingi, V. *et al*. Stability and dynamics of membrane-spanning DNA nanopores. *Nat. Commun.* **8,** 14784 doi: 10.1038/ncomms14784 (2017).

**Publisher's note**: Springer Nature remains neutral with regard to jurisdictional claims in published maps and institutional affiliations.

## Supplementary Material

Supplementary Information Supplementary Figures, Supplementary Table, Supplementary Methods and Supplementary References.

Supplementary Movie 1 This is a movie version of Figure 5d, as explained in the main text.

## Figures and Tables

**Table 1:** Summary of simulations performed.

**Simulation**	**Duration**
*CG simulations*	
DNT^Et^PC*	6 × 1 μs
iDNT^Et^PC†	6 × 1 μs
iDNT^Et^PCold‡	4 × 1 μs
DNT^Et^PCsoft§	6 × 1 μs
DNT^Et^PC/PS||	6 × 1 μs
DNT^Et^PE/PG¶	6 × 1 μs
DNT^Et^-PM#	6 × 1 μs
DNT^6Et^PC**	6 × 1 μs
DNT^1Et^PC††	6 × 1 μs+2 × 10 μs
DNT^2AEt^PC‡‡	3 × 1 μs
DNT^2DEt^PC§§	3 × 1 μs
DNT^nEt^PC||||	6 × 1 μs
5 displaced DNT^Et^PCs (see Fig. 3)	1 μs each
SMD (push in) DNT^Et^PC (see Fig. 5a,b)	10 nm μs^−1^; >2 μs × 3
SMD (push in) DNT PC (see Fig. 5c)	10 nm μs^−1^; >2 μs × 3
SMD (push in) DNT^Et^PCxy¶¶ (see Supplementary Fig. 6A)	10 nm μs^−1^; >2 μs
SMD (push in) DNT PCxy¶¶ (see Supplementary Fig. 6A)	10 nm μs^−1^; >2 μs
SMD (pull out) DNT^Et^PC (see Supplementary Fig. 7)	10 nm μs^−1^; >1.5 μs
SMD (push in) DNT^1Et^PC (see Supplementary Fig. 6B)	10 nm μs^−1^; >2 μs × 2
SMD (push in) DNT^2AEt^PC (see Supplementary Fig. 6C)	10 nm μs^−1^; >2 μs × 2
	
*AT simulations*	
DNT^6Et^PC, 0.15 M NaCl	0.5 μs
DNT^6Et^PC, 1.0 M NaCl	0.5 μs

CG, coarse grain; DNT, DNA nanotube; ENM, elastic network model; PC, phosphatidylcholine; PE, phosphatidylethanolamine; PG, phosphatidylglycerol; PS, phosphatidylserine; SMD, steered molecular dynamics.

^*^This is the main CG simulation—DNT^Et^PC with seven hydrophobic bands (see Supplementary Fig. 1). All CG simulations were performed using 1.0 M NaCl.

^†^i=initial DNT model used^27^.

^‡^old=MARTINI version^76^ used for lipids.

^§^soft=soft ENM used for DNT.

^||^PC/PS=lipids a mixture of 80% PC and 20% PS.

^¶^PE/PG=lipids a mixture of 75% PE and 25% PG.

^#^PM=lipids from a model plasma membrane (see ref. 51 and Supplementary Fig. 3 for details).

^**^6Et=6 hydrophobic bands instead of 7 in CG or atomistic (AT) simulations (see Supplementary Fig. 1).

^††^1Et=single-hydrophobic band (see Supplementary Fig. 1).

^‡‡^2AEt=2 adjacent-hydrophobic bands (see Supplementary Fig. 1).

^§§^2DEt=2 distal-hydrophobic bands (see Supplementary Fig. 1).

^||||^nEt=an extended-hydrophobic patch covering the entire DNT surface (see Supplementary Fig. 10).

^¶¶^Positional restraints in the *xy* plane used in the SMD to keep the DNT-long axis parallel to the bilayer normal (see Supplementary Fig. 6A).
