# Supplementary material for: Stability and dynamics of membrane-spanning DNA nanopores
Source: Nat Commun. 2017 Mar 20;8:14784. doi: 10.1038/ncomms14784 (PMC5364398; doi:10.1038/ncomms14784)
Supplement: Supplementary Information — Supplementary Figures, Supplementary Table, Supplementary Methods and Supplementary References. [file ncomms14784-s1.pdf]

Supplementary Methods

Structures for simulations

Our coarse-grain (CG) simulations started from an atomistic (AT) DNA nanotube (DNT) model described previously, using cluster analysis [1] to select a structure from simulation DNT1 described in Ref. [2].

The AT structures were coarse-grained using *martinize.py* [3]. We modified this script for DNA-origami structures in order to create stiff elastic network for the DNT, acting between each double helix and between the staples connecting the helices. The CG models were provided with hydrophobic modifications corresponding to ethyl groups (Supplementary Figure 1).

Steered molecular dynamics CG simulations

A CG POPC bilayer was simulated for 200 ns, and final snapshot was used as a starting point for steered molecular dynamics (MD) simulations. A DNT molecule (DNT, DNT^{Et}, DNT^{2AEt} or DNT^{1Et}: see Table 1 for details, and abbreviations used) was placed above the membrane, with the minimum distance between POPC and DNT set to ~3.2 nm. The distance between the centers of mass (COM) of the membrane and of the hydrophobic in the DNT was initially ~12 nm. This simulation box size thus was ~22 x 22 x 34 nm^3, and the bilayer/DNT system was solvated with water particles and 1 M NaCl. Positional restraints of 1000 kJ mol^{-1} nm^{-2} were applied to the DNT for 50 ns to equilibrate the water and ions around DNA and membrane. Positional restraints on the DNT were then removed, and steered MD was performed by pulling, at the rate of 10 nm μs^{-1}, the COM of the modified groups in the DNT along the *Z* axis relative to the COM of membrane. The pulling force constant was set to 1000 kJ mol^{-1} nm^{-2}. Three repeats for each case simulation were performed with different random seeds for initial velocities. Using the same initial set ups, additional steered MD simulations were conducted by applying 100 kJ mol^{-1} nm^{-2} positional restraints in the *X* and *Y* dimensions on the DNT particles to avoid tumbling of the nanotube relative to the *Z*-axis. The corresponding plots are shown in Supplementary Figure 6 and Figure 5. For steered MD simulations *NVT* conditions were employed.

A further steered MD simulation was performed in which an initially embedded DNT^{Et} nanopore was pulled out of the lipid bilayer. The initial configuration was obtained from a 1 μs CG simulation of DNT^{Et}PC. The resultant plot is shown in Supplementary Figure 7.

Experimental studies

Materials

Native and cholesterol-labeled DNA oligonucleotides were purchased from Integrated DNA Technologies (Iowa US) or ATD Bio (UK), on a 1 µmol scale with HPLC or PAGE purification. 1,2-Diphytanoyl-*sn*-glycero-3-phosphocholine (DPhPC) was procured from Avanti Polar Lipids. n-Octyl-oligo-oxyethylene was purchased from Enzo Life Sciences (UK). All other reagents and solvents were purchased from Sigma-Aldrich.

Sequences of component DNA strands of DNA nanopores

Sequences for the DNA strands forming DNA nanopore with cholesterol anchors (DNT^{2C}) are shown in Supplementary Table 1, and the 2D map of the corresponding DNA nanopore is shown in Supplementary Figure 11.

Supplementary Table 1. Sequences of DNA strands used for DNT^{2C}. See also Supplementary Figure 11.

ID	Sequence 5'→ 3'
1	ACAGGATTTTCGCCTGCTGGGGCAAACCAGCGTGGACCGCTTTTTT GGCTATTCTTTTGATTTATAAGGGATTTTGCCGATTTCGGAA
2	CAACTCTCTCAGGGCCAGGCGGTGAAGGGCAATCAGCTGTTGTTTT CAACAGCATCCTGTTTCCGAAATCGGCATTAAAGACCAGCTG
3	TCTCACTGGTGAAAAGAAAAACCACCCTGGCGCCCAATACGCTTTTT CCCCGCGCGTTGGCCGATTCATTAATGCAGCTGGCACGACA
4	GGCGAAATGATTGCTTTCACCAGTGAGATGTCGTGACGTGGATTTTT CCACGTTCTTTAATAGTGGACTCTTGTTCCAAACTGGAACA
5	TGTTCCAAATAGCCAAGCGGTCCACGCTCCCTGAGGGGCGCC
6	5'tri(ethylene glycol)-chol/CATTAATTTTTTCTCCTTCACCGCCTGGGGTTTGCTTATAAA
7	AGGGTGGGAATCGGACAAGAGTCCACTAAAATCCCCCCAGCA 3'tri(ethylene glycol)-chol
8	TCAAAAGGTTTGGACCAACGCGCGGGGAGCGTATTAGAGTTG

Folding of DNA nanopores

An equimolar mixture of DNA oligonucleotides 1 to 8 (1 nmol each, dissolved in 1 M KCl, 50 mM Tris pH 8.0; total volume 1,000 µl) was prepared at room temperature, incubated at 95 °C for 2 min and cooled to 20 °C at a rate of 0.5 °C per min using a PCR thermocyler.

Agarose gel electrophoretic analysis of DNA nanopore (DNT^{2C})

See the corresponding analysis in Supplementary Figure 12.

Nanopore current recordings

Electrophysiological current measurements was performed following a modified version of a published protocol [4]. Using a chip-based, parallel bilayer recording set-up (Orbit 16, Nanion Technologies, Munich, Germany) with multi-electrode-cavity-array (MECA) chips (Ionera Technologies, DE), and electrolyte solution (150 µL, 1 M KCl, 10 mM HEPES pH 8.0 or 0.3 M KCl, 10 mM HEPES pH 8.0). Bilayers were automatically formed by remotely actuated spreading of DPhPC dissolved in hexane (1 mg ml^{-1}).

For pore insertion, a 2:1 mixture of DNT^{2C} and 0.5% n-octyl-oligo-oxyethylene dissolved in (1 M KCl or 0.3 M KCl, 10 mM HEPES pH 8.0) was added to the *cis* side of the bilayer to a final concentration of 10 nM nanopores. A positive voltage of +50 or +100 mV was applied to facilitate pore insertion. The ionic current data were Bessel filtered at 2.07447 kHz and acquired at 20 kHz using an EPC-10 patch-clamp amplifier (HEKA Elektronik, DE) with the PATCHMASTER software (HEKA Elektronik, DE). Single channel analysis was performed using Clampfit (Molecular Devices, CA, USA).

Melting temperature determination

The melting temperature of the cholesterol modified nanopore incorporated into DPhPC small unilamellar vesicles was determined by UV-vis absorption spectroscopy as described in [4].

Supplementary Figures

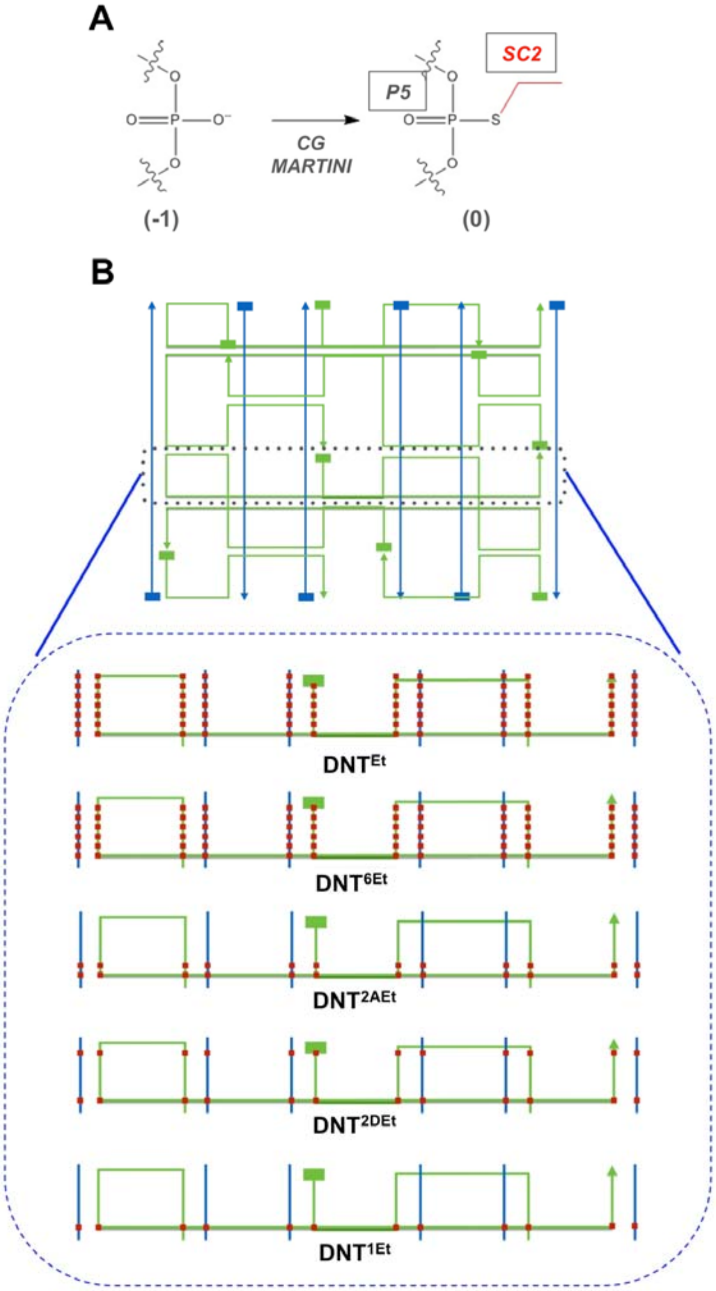

Supplementary Figure 1. Topology and positioning of hydrophobic particles in the DNA nanotube model

(A) Schematic diagram showing the ethyl modification of the DNA backbone. Boxes on the right hand side diagram indicate the MARTINI particle types used for coarse-graining. **(B)** The upper panel shows the six scaffold strands (blue) and the eight staple strands of the DNA nanotube (design taken from [5]). The lower panel shows the positions of the hydrophobic ethyl (Et; in red) modifications in the different models discussed in Table 1 of the main text.

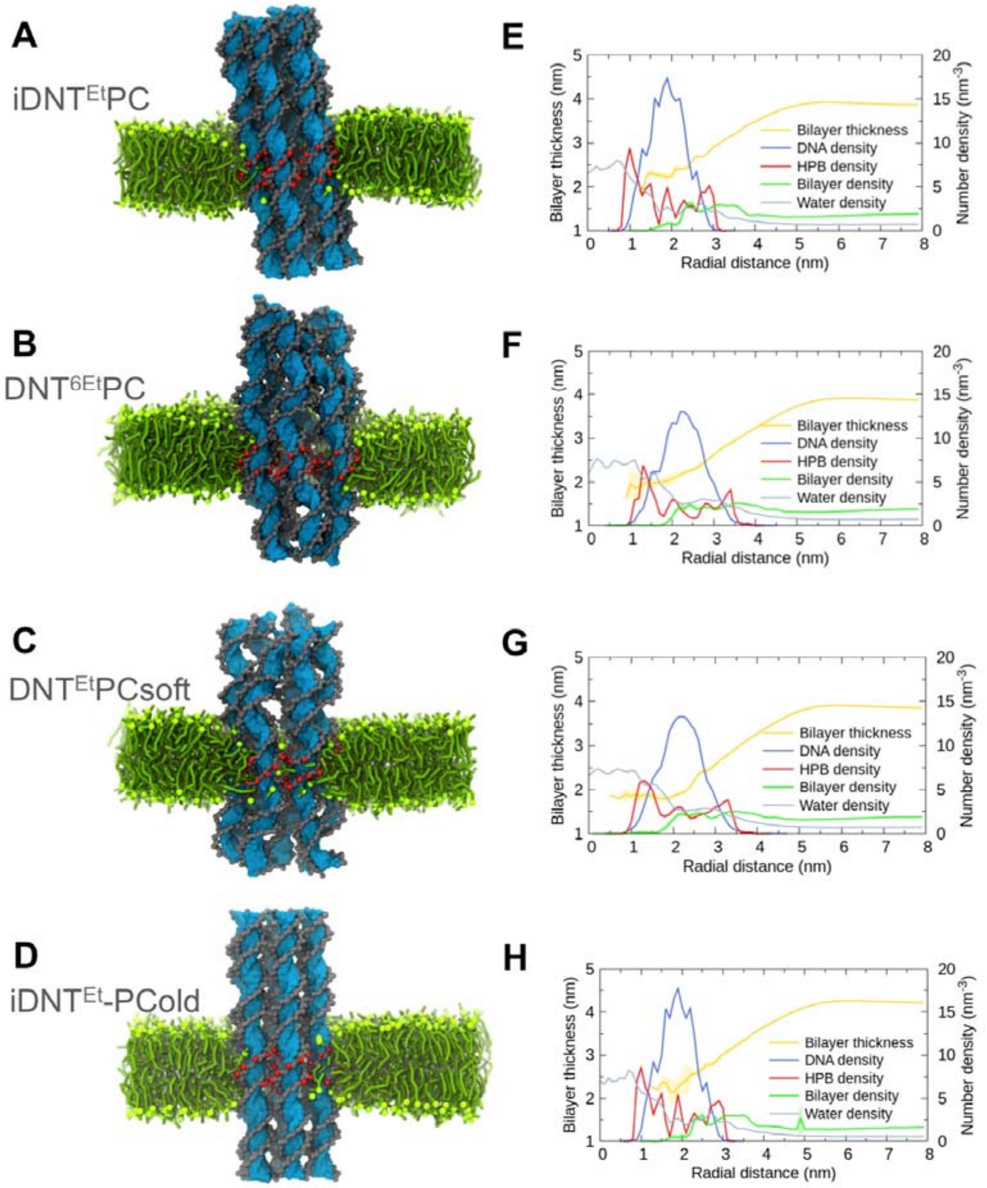

Supplementary Figure 2. **A range of DNT models**

(A-D) Final snapshots showing different DNT models embedded in a membrane. See Table 1 for summary of simulations performed, and abbreviations used. *Red beads*, hydrophobic modifications (HPB); *blue surface*, DNA bases; *grey surface*, DNA backbone; *green sticks/beads*, POPC/phosphate head group. **(E-H)** Bilayer thickness profiles around the embedded DNTs along with the radial density distribution of DNA-phosphates, hydrophobic particles, bilayer phosphates, and water particles. Note that the lipid bilayer and hydrophobic particle (i.e. ethyl; Et group) densities have been scaled up 5x for ease of visualization.

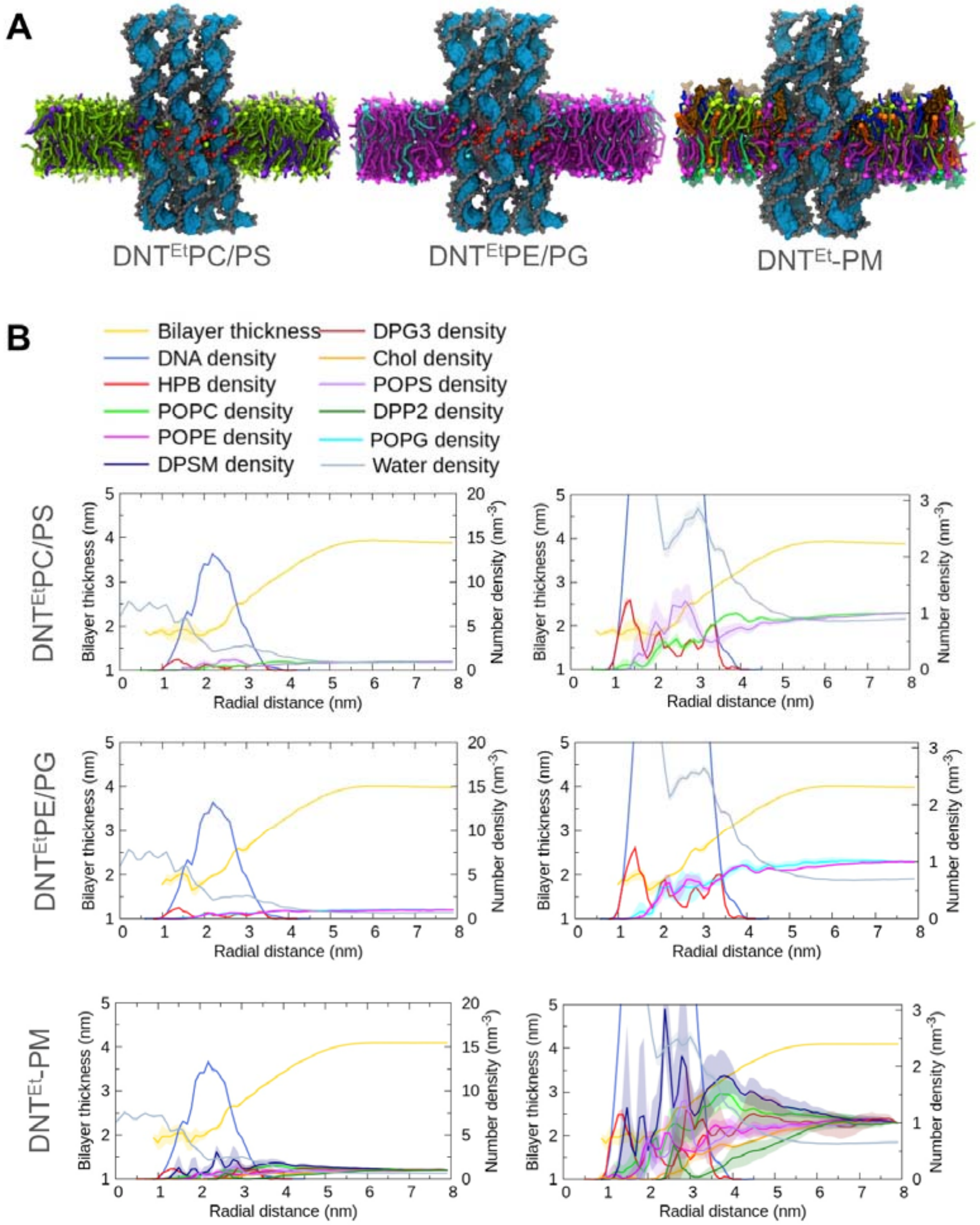

Supplementary Figure 3. DNT^{Et} interaction with mixed lipid bilayer membranes
(A) Final snapshots showing DNT^{Et} embedded in membranes with different lipid compositions. See Table 1 for summary of simulations performed, and abbreviations used. *Red beads*, hydrophobic modifications (HPB); *blue surface*, DNA bases; *grey surface*, DNA backbone. Lipid colours (beads, sticks or surface view) correspond to the number density plot colours as shown in **B**. **(B)** Left panel shows bilayer thickness profiles around the embedded DNTs along with the radial density distribution of DNA-phosphates, hydrophobic particles, bilayer beads ('PO4' or 'AM1' or 'ROH' MARTINI beads depending upon lipid type), and water particles. Right panel shows the corresponding magnified density profiles along with bilayer thickness profiles. Lipid compositions are as follows: POPC:POPS both leaflets (80:20); bacterial membrane POPE:POPG both leaflets (75:25); Plasma membrane POPC:POPE:DPSM (sphingomyelin):DPG3 (GM3):Chol outer leaflet (40:10:15:10:25) and POPC:POPE:POPS:DPP2 (PIP2):Chol inner leaflet (10:40:15:10:25).

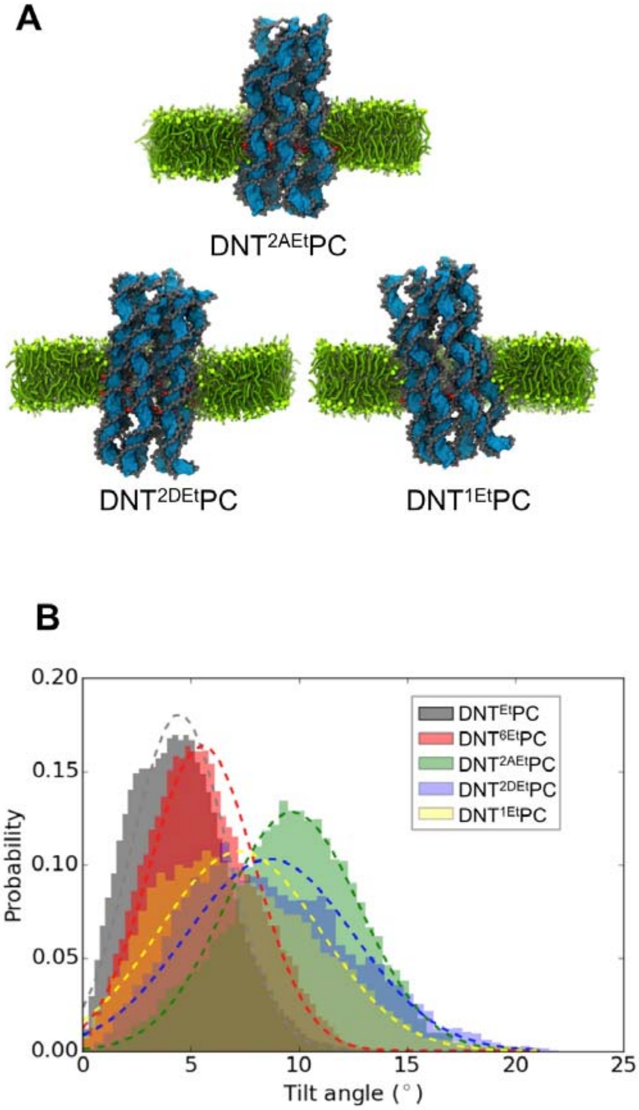

Supplementary Figure 4. Varying the extent of the hydrophobic anchor surface
(A) Final (1 μs) snapshots are shown for various simulation systems of DNA nanotubes (DNTs) having different number of ethyl modifications (2x12; adjacent and distal, 1x12). **(B)** DNT tilt angle distributions for the different DNT simulation systems shown in **A.** Tilt was calculated as the angle between the *Z*-axis (i.e. approximate bilayer normal) and the long axis passing through the center of each DNT.

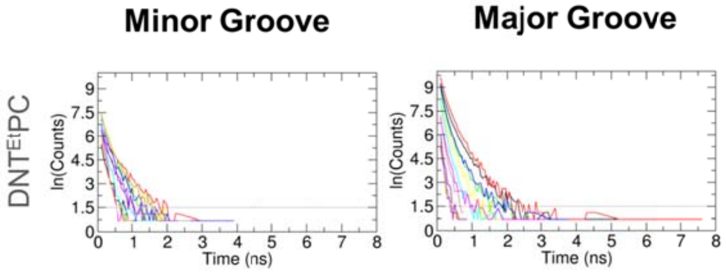

Supplementary Figure 5: **Mean residence times of lipids in the DNA surface grooves** Residence time distributions for lipids in the minor and major grooves of the DNA nanotube. Residence contact times, with major or minor grooves, were calculated only for those lipids interacting with the nanotube. Mean residence times were calculated as inverse of slopes obtained from linear regression fitting of a simple exponential decay function.

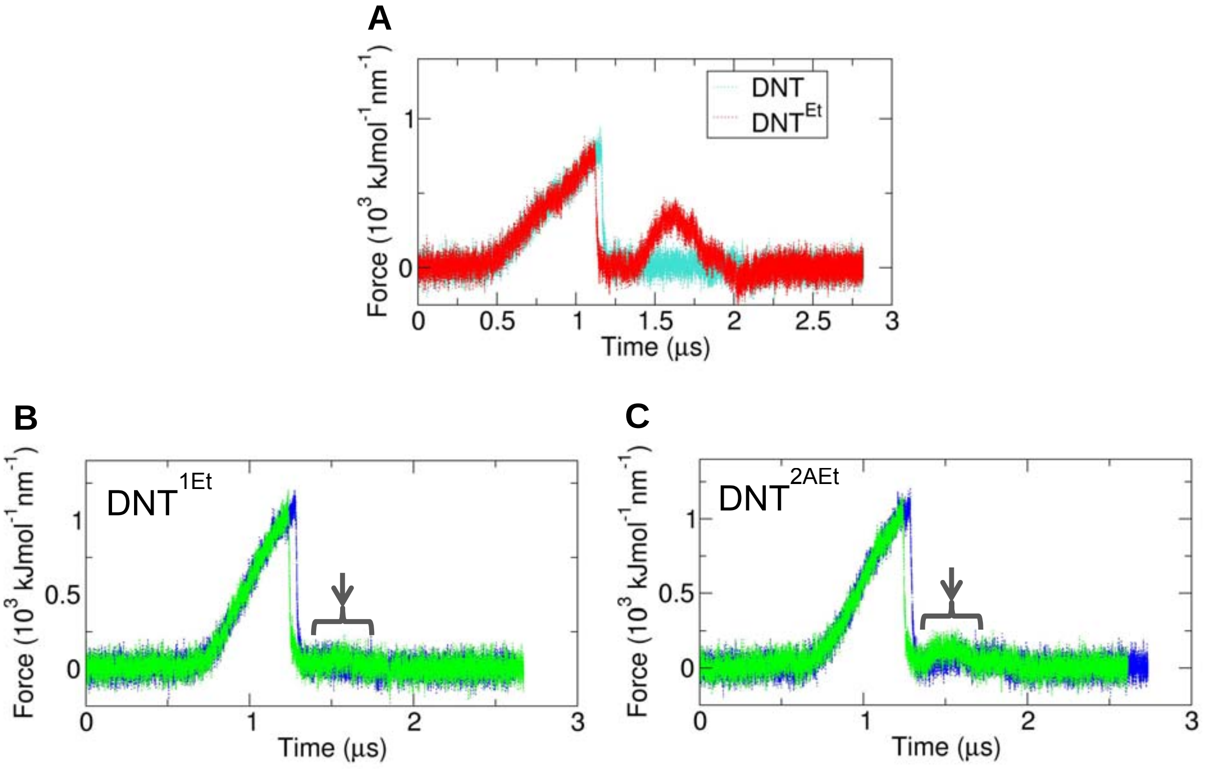

Supplementary Figure 6: Steered MD simulations (push in)

(A) Force *vs* time curves shown for DNT (cyan) and DNT^{Et} (red) steered through a POPC bilayer with positional restraints in *XY* to avoid DNT tumbling (see main text and Figure 5 for details). **(B)**, **(C)** Force *vs*. time curves for steered simulations of DNT^{1Et} and DNT^{2AEt}. Arrows highlight low barriers for DNT^{1Et} and DNT^{2AEt} removal, which is in sharp contrast to the DNT^{Et} case (Figure 5). Repeat simulations are shown in blue and green.

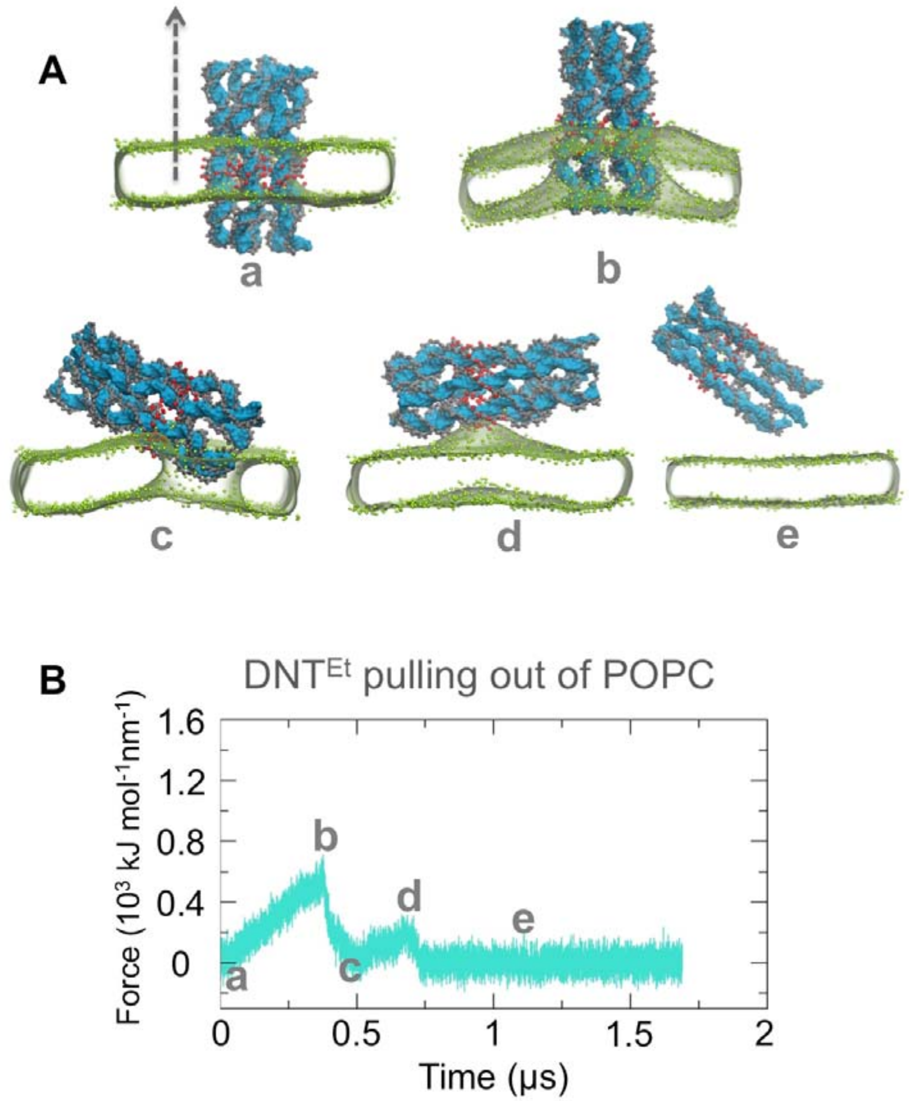

Supplementary Figure 7. **Steered MD simulations (pull out)**

(A) Snapshots *a-e* showing DNT^{Et} steered (i.e. pulled) out from an embedded state at a rate of 10 nm μs^{-1}. **(B)** Force *vs* time curve with the labels *a* to *e* corresponding to the snapshots in **A**.

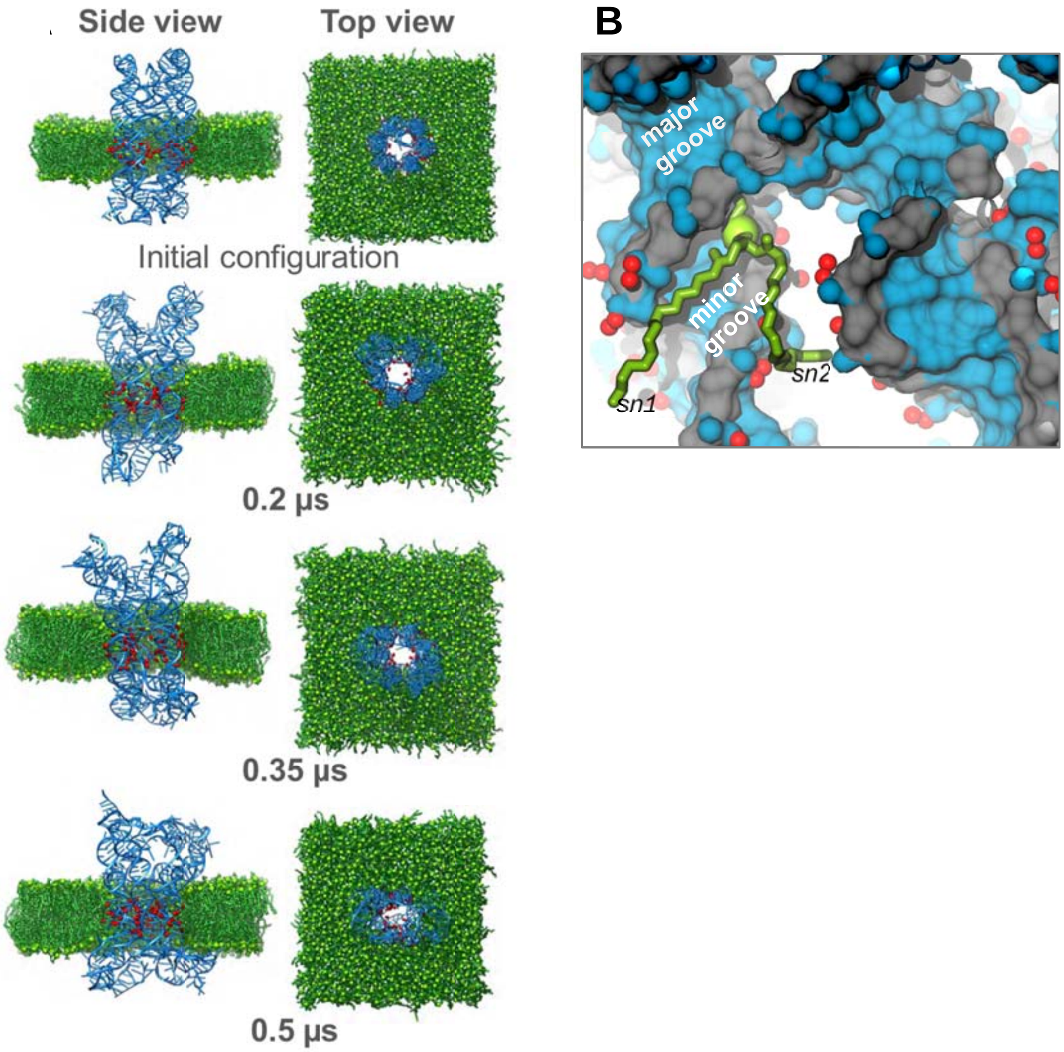

Supplementary Figure 8. All atom DNT-membrane simulations
(A) Successive snapshots are shown for the AT $DNT^{6Et}PC$ simulation (see Table 1 for abbreviations). Ethyl groups are shown as *red* spheres, DNA is represented as a *blue* ladder, POPC molecules are shown in *green*. This simulation was performed in 0.15 M NaCl (waters, ions are not shown for clarity). **(B)** Snapshot showing a selected lipid, the headgroup and *sn1* acyl chain of which interact with the minor groove surface of for the AT-DNT^{6Et} PC simulation in 1.0 M NaCl.

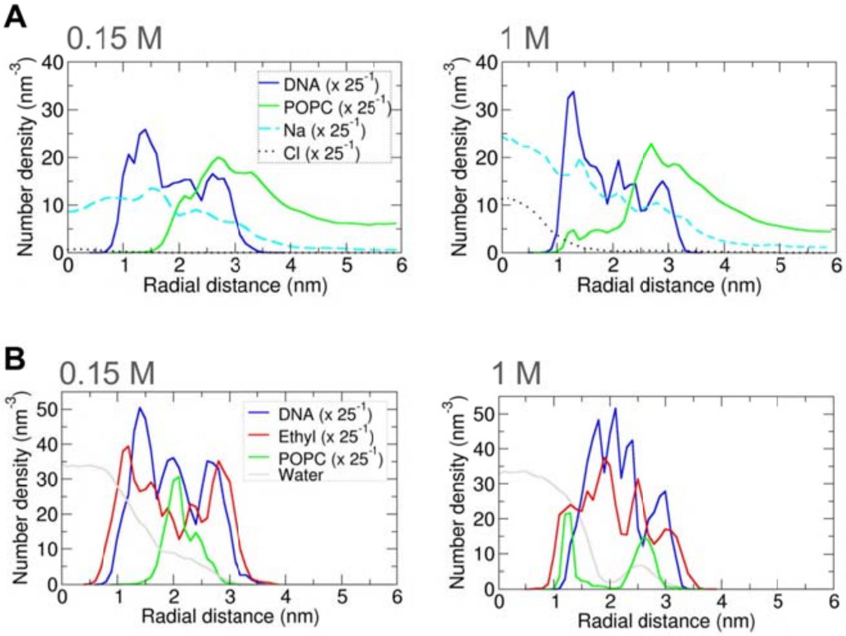

Supplementary Figure 9. Radial density distributions

(A) Radial density distribution of DNA phosphates, lipid phosphates and ions for the AT DNT^{6Et} simulations at 0.15 and 1.0 M NaCl (see Table 1 for abbreviations used). These distributions correspond to a cylinder of height 4.1 nm which therefore extends from the centre of the bilayer just beyond the headgroup region of the lipids on each side. **(B)** Radial density distribution of DNA phosphates, ethyl group terminal carbons, lipid phosphates and waters for the same two simulations. These distributions correspond to a cylinder of height 0.5 nm which therefore cover the central region of the hydrophobic core of the bilayer.

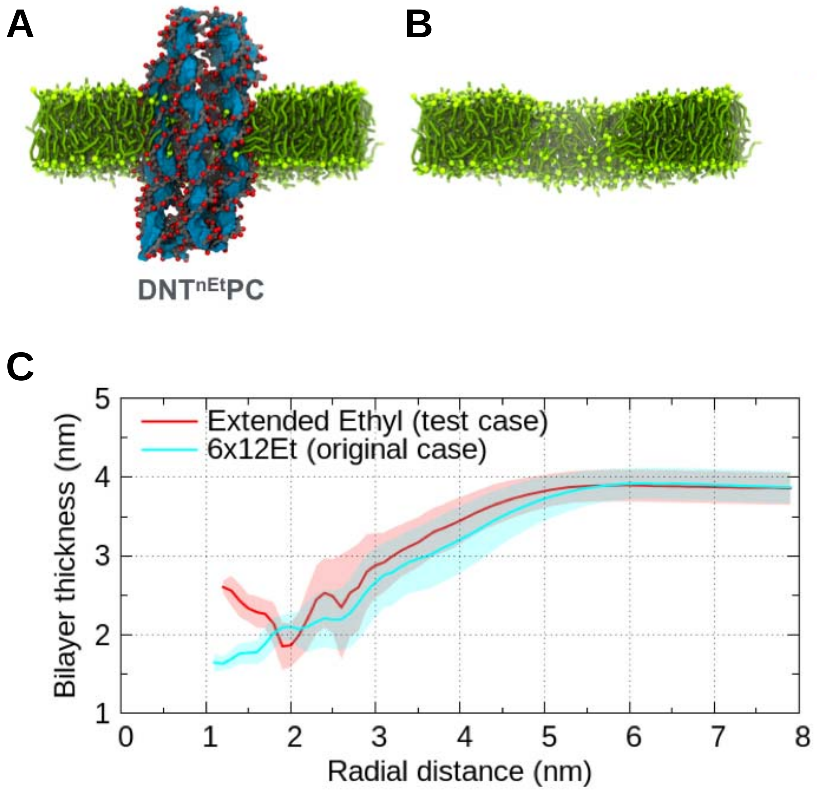

Supplementary Figure 10. **DNT with an extended hydrophobic patch**

(A) Final snapshot showing a DNT^{nEt} model with an extended hydrophobic patch (covering the entire DNT surface) embedded in a PC membrane. See Table 1 for summary of simulations performed, and abbreviations used. *Red beads*, hydrophobic modifications; *blue surface*, DNA bases; *grey surface*, DNA backbone; *green sticks/beads*, POPC/phosphate head group. (**B**) View of the same system, showing only the lipids and revealing the local perturbation of the bilayer by the inserted DNA nanopore. **(C)** Bilayer thickness profiles comparison around the embedded DNTs.

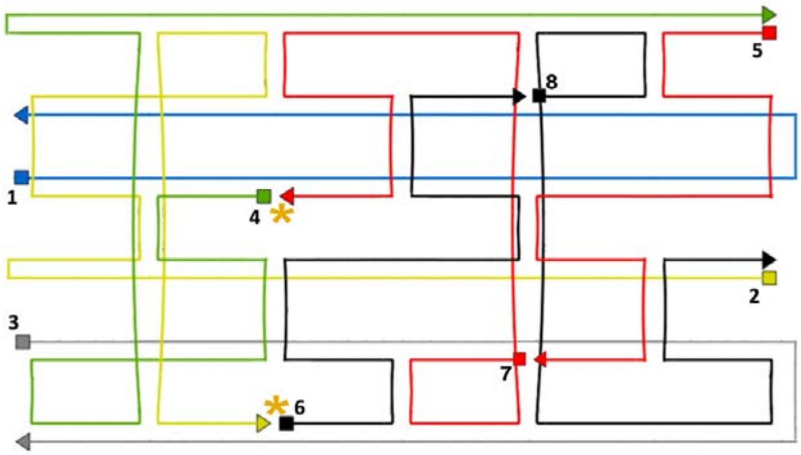

Supplementary Figure 11. **2D map of DNA nanopore (DNT^{2C})**
2D map of cholesterol-modified DNA nanotube (DNT^{2C}) is based on a previously published structure [6]. The pore is composed of DNA strands 1 to 8, indicated by numbers (See Supplementary Table 1 also). Strands 6 and 7 contain 5' and 3' terminal tetra-ethylene glycol cholesterol modifications, respectively. The positions of the cholesterol anchors are highlighted by orange asterisks.

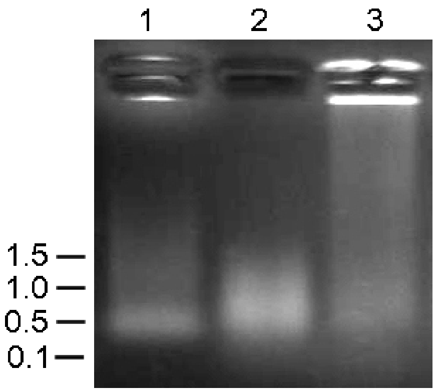

Supplementary Figure 12. Agarose gel electrophoretic analysis

1.2% agarose gel analysis of DNA nanopore and control nanostructures visualized by ethidium bromide staining. Lane 1, control pore without hydrophobic groups; lane 2, DNA nanopore carrying alternative hydrophobic anchors composed of ethylated phosphorothioate [6]; lane 3, cholesterol-modified pore (DNT^{2C}). The kilobase pair bands are annotated on the left of the gel, established from a 100 bp marker from New England Biolabs (UK).

Supplementary References

1 Daura, X. *et al.* Peptide folding: When simulation meets experiment. *Angewandte Chemie-International Edition* **38**, 236-240 (1999).

2 Maingi, V., Lelimousin, M., Howorka, S. & Sansom, M. S. P. Gating-like motions and wall porosity in a DNA nanopore scaffold revealed by molecular simulations. *ACS Nano* **9**, 11209–11217 (2015).

3 Uusitalo, J. J., Ingolfsson, H. I., Akhshi, P., Tieleman, D. P. & Marrink, S. J. Martini coarse-grained force field: extension to DNA. *J. Chem. Theor. Comput.* **11**, 3932-3945 (2015).

4 Burns, J. R., Seifert, A., Fertig, N. & Howorka, S. A biomimetic DNA-based channel for the ligand-controlled transport of charged molecular cargo across a biological membrane. *Nature Nanotech.* **11**, 152-156 (2016).

5 Burns, J. R., Stulz, E. & Howorka, S. Self-assembled DNA nanopores that span lipid bilayers. *Nano Lett.* **13**, 2351-2356 (2013).

6 Burns, J. R., Al-Juffali, N., Janes, S. M. & Howorka, S. Membrane-spanning DNA nanopores with cytotoxic effect. *Angew. Chem. Int. Ed.* **53**, 12466-12470 (2014).
